# Regulation of lamin properties and functions: does phosphorylation do it all?

**DOI:** 10.1098/rsob.150094

**Published:** 2015-11-18

**Authors:** Magdalena Machowska, Katarzyna Piekarowicz, Ryszard Rzepecki

**Affiliations:** Laboratory of Nuclear Proteins, Faculty of Biotechnology, University of Wrocław, ul. Fryderyka Joliot-Curie 14a, Wrocław 50–383, Poland

**Keywords:** nuclear envelope, lamin polymerization, chromatin binding, kinase motif, Cdk1/PKA/PKC, signalling

## Abstract

The main functions of lamins are their mechanical and structural roles as major building blocks of the karyoskeleton. They are also involved in chromatin structure regulation, gene expression, intracellular signalling pathway modulation and development. All essential lamin functions seem to depend on their capacity for assembly or disassembly after the receipt of specific signals, and after specific, selective and precisely regulated interactions through their various domains. Reversible phosphorylation of lamins is crucial for their functions, so it is important to understand how lamin polymerization and interactions are modulated, and which sequences may undergo such modifications. This review combines experimental data with results of our *in silico* analyses focused on lamin phosphorylation in model organisms to show the presence of evolutionarily conserved sequences and to indicate specific *in vivo* phosphorylations that affect particular functions.

## 1. Introduction

**Figure 1.**
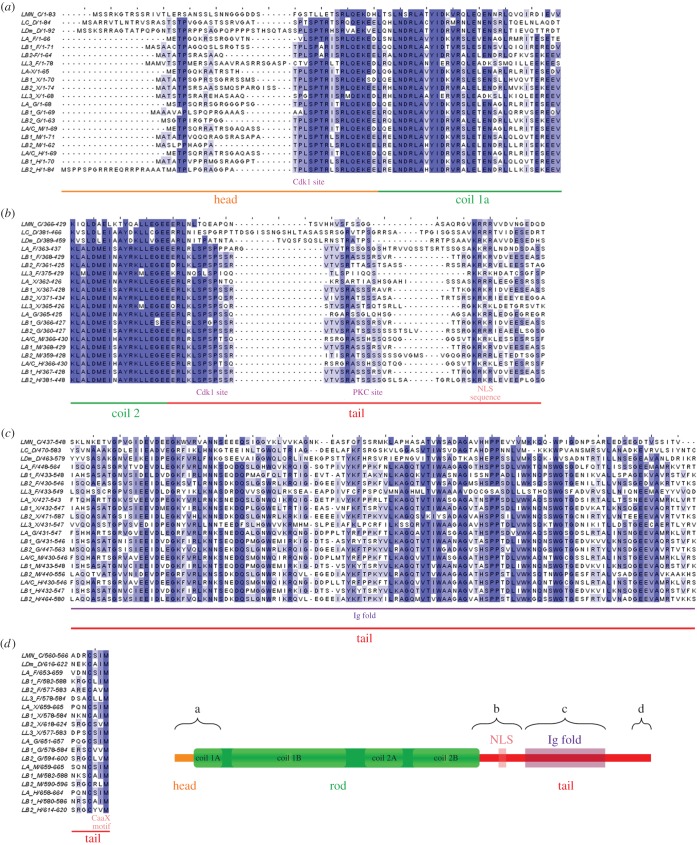
Multiple sequence alignment of selected lamins of all types. Lamins from different species were chosen: lamin from *Caenorhabditis elegans* (LMN_C), lamins A, B1, B2 and L3 from *Danio rerio* (LA_F, LB1_F, LB2_F, LL3_F), lamins A, B1, B2 and L3 from *Xenopus laevis* (LA_X, LB1_X, LB2_X, LL3_X), lamins A, B1 and B2 from *Gallus gallus* (LA_G, LB1_G, LB2_G), lamins A, B1 and B2 from *Mus musculus* (LA_M, LB1_M, LB2_M), and lamins A, B1 and B2 from *Homo sapiens* (LA_H, LB1_H, LB2_H). Sequences of four selected fragments of proteins were aligned and analysed: (*a*) the N-terminus end (the head domain and coiled-coil region 1a of the rod domain); (*b*) the last 20 amino acids of coiled-coil region 2 of the rod domain and the first part of the tail domain; (*c*) the Ig fold region; and (*d*) the C-terminus end (only farnesylated lamins). Selected fragments of murine and human lamins A and C analysed in panels (*a*), (*b*) and (*c*) have identical sequences. The darker blue colour indicates higher similarity (also in figures 2–5).

The evolutionarily oldest members of the intermediate filament (IF) protein family, the lamins are type V IF proteins. All the discovered lamins share the same overall domain structure independently of the organism of origin [1,2]. The structure has a central rod domain flanked by a short N-terminal head domain and a long C-terminal tail domain (figure 1). The central rod domain consists of four coiled-coil segments separated by flexible linker regions. The tail domain contains several conserved motifs, including a nuclear localization signal (NLS), a globular immunoglobulin fold (Ig fold) [3] and a consensus signal sequence for farnesylation known as the CaaX box (cysteine residue, two aliphatic amino acid residues and any aminoacid residue, which in lamins is always methionine) [4].

Polymerization of lamins is essential for their mechanical functions, and all the domains are involved in this process. The head and tail domains contain two conservative motifs called ‘mitotic’ phosphorylation sites (cdc2/Cdk1 sites) that are crucial for the regulation of nuclear lamina and lamin disassembly in most metazoans [5]. Lamins form dimers that associate longitudinally, creating head-to-tail polymers, then assemble in anti-parallel protofilaments, three to four of which associate laterally to form IFs [6].

In general, two major types of lamin are distinguished: A-type and B-type. The number of genes for lamins increased during the evolution of the metazoans. *Caenorhabditis elegans* has only a single gene coding for a single Ce-lamin protein, which functionally resembles both A- and B-type lamins of vertebrates, although it lacks some of the typical structural features of vertebrate B-type lamins. In *Drosophila melanogaster*, two lamin genes are present, coding for lamin Dm (resembling B-type lamins) and lamin C (resembling A-type lamins) [7–9].

Mammals typically have three lamin genes, coding for lamins A/C, B1 and B2. As a result of alternative splicing, more than three protein products are synthesized. The *LMNA* gene codes for lamins A (and AΔ10), C and the additional variant C2, which is mainly produced in the reproductive cells*.* The *LMNB1* gene codes for lamin B1, and *LMNB2* codes for lamins B2 and B3 [10].

A more complex system of lamin genes and proteins exists in other vertebrates. In teleost fish, amphibians and birds, apart from the genes coding for lamins A (with no splicing to lamin C), B1 (LI) and B2 (LII), there is also a gene coding for lamin B3 (LIII), the expression of which is almost exclusive to the oocyte and early embryo. The lamin B3 gene in amphibians codes for three proteins due to alternative splicing: lamins B3a (LIIIa), B3b (LIIIb) and LIV [10]. There is also additional lamin B3 (LIII) in chicken, and some fish species have an additional lamin A [11].

The unquestioned main role of lamins is their mechanical and structural function as major building blocks of the karyoskeleton. Lamins A and C have also been implicated in the regulation of chromatin structure, intracellular signalling pathways, stiffness, plasticity and development [1,12]. Reversible polymerization is not only required for the structural role of lamins, but also for some regulatory functions, which often rely on targeting to specific locations or the immobilization of interacting protein complexes. For instance, lamins are involved in the positioning and regulation of pRb protein through its binding with LAP2*α* [13–16] (for review see [17]), binding of transcription factor MOK2 [18] and binding to c-FOS [19]. Specific phosphorylation patterns of lamin protein particles interacting with proteins of interest may translocate or make such proteins mobile. Another example of the localization-dependent function of lamins is the positioning of specific proteins or protein complexes, such as transcription factors, chromatin remodelling complexes, or nucleosomes with specific subsets of histone variants and modifications [20,21].

As lamin functions depend on the capacity for assembly or disassembly and specific, precisely regulated interactions, it is crucial to understand which domain of the lamins is responsible for polymerization and binding partners, and how the processes are modulated. It is widely known that lamins undergo post-translational modifications via phosphorylation, farnesylation, myristoylation, ubiquitination, acetylation, sumoylation and proteolytic cleavage [22]. The only modification that is easily reversible and common for all lamins is phosphorylation. It allows for flexible activation or inactivation of all lamin functions and interactions.

Our study focused on the conserved domains of particular lamins, their function and their biological effect depending on these reversible reactions. The frequency of discovered phosphorylation is significantly higher in the head and tail domain than in the rod domain, so we principally investigated these two regulatory domains.

**Figure 2.**
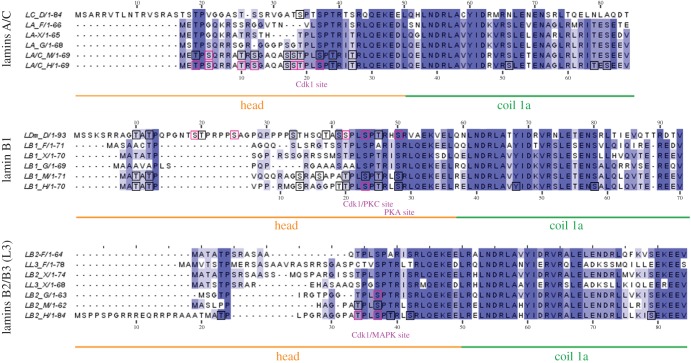
Multiple sequence alignment of N-terminus end of selected lamins. The N-terminus end (the head domain and coiled-coil 1a) of selected lamins were aligned and analysed separately in three groups according to the gene of origin: lamins A/C, lamin B1 and lamins B2/B3 (L3). Black frames mark particular phosphosites identified in large-scale proteomics studies only, while pink frames mark sites mapped both individually in lamin-specific experiments and in large-scale proteomics studies (also in figures 3–5). The numbering below the alignments refers to human lamins. The numbering above lamins A refers to *D. melanogaster* lamin C and above B1 lamins refers to lamin Dm. All the kinases presented below the alignments were confirmed experimentally and exact phosphorylated residues are shown in table 1 (also in figures 3–5).

**Figure 3.**
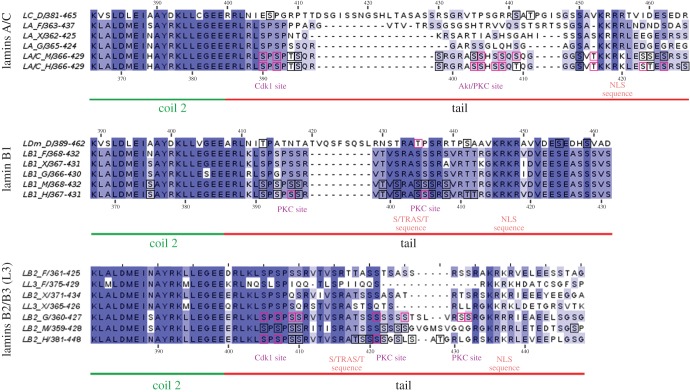
Multiple sequence alignment of the last 20 amino acids of the rod domain and initial region of the tail domain of selected lamins. Sequences were aligned and analysed separately in three groups according to the gene of origin: lamins A/C, lamin B1 and lamins B2/B3 (L3).

**Figure 4.**
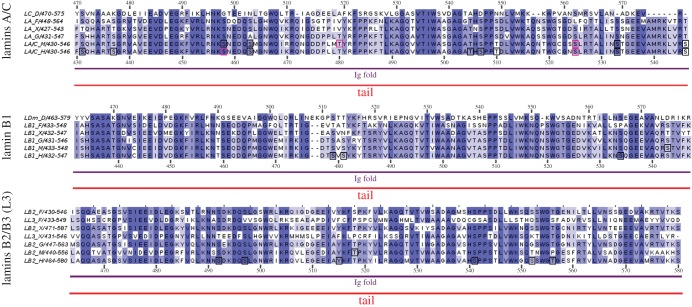
Multiple sequence alignment of the Ig fold region of selected lamins. The Ig fold region of selected lamins was aligned and analysed separately in three groups according to the gene of origin: lamins A/C, lamin B1 and lamins B2/B3 (L3).

**Figure 5.**
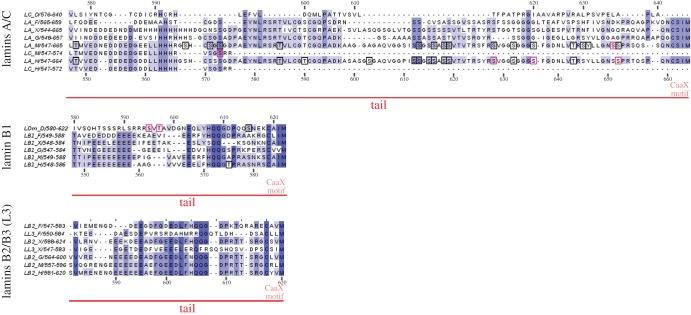
Multiple sequence alignment of the C-terminus end of selected lamins. The C-terminus end (the entire sequence after the Ig fold) of selected lamins was aligned and analysed separately in three groups according to the gene of origin: lamins A/C, lamin B1 and lamins B2/B3 (L3).

**Figure 6.**
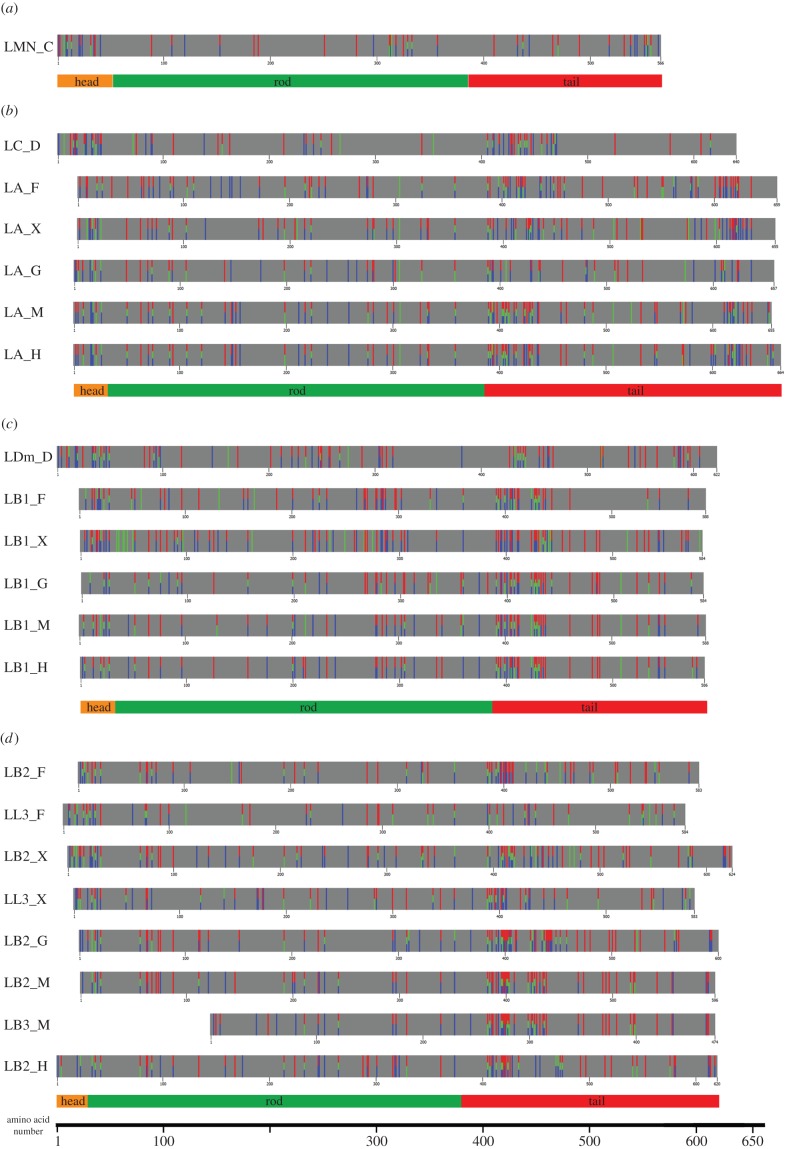
Predicted phosphorylation sites of selected lamins: (*a*) *C. elegans* lamin, (*b*) lamins A/C, (*c*) lamin B1 and (*d*) lamins B2/B3 (L3). Entire sequences of lamins were analysed using three different programs for phosphorylation site prediction: NetPhos (red), KinasePhos (green) and DISPHOS (blue). Bars were aligned with their rod domains. The scale under the bars indicates the number of amino acids. The caption for the head, rod and tail domains on the scheme are scaled to human lamin in each group (except *C. elegans* lamin).

Only model organisms were used for studying lamins in this work, despite some of them not being the most representative species of their systematic groups. The reason was to have models with the most available functional data. Moreover, our data will facilitate future functional investigations, which are predominantly performed on model organisms. We aligned the essential lamins protein sequences of *C. elegans*, *D. melanogaster*, *Danio rerio*, *Xenopus laevis*, *Gallus gallus*, *Mus musculus* and *Homo sapiens*, and marked conservative, specific sequences, both within all the analysed lamins together (figure 1) and among particular types (figures 2–5). We also analysed the available data concerning identified phosphosites, dividing them in two groups: lamin-specific experiments (pink frames in figures 2–5) and large-scale proteomics studies performed with mass spectrometry (black frames in figures 2–5). As large-scale analysis can generate false-positive results, we graduated the available records depending on how many times a particular phosphorylation site was identified, so we indicated the most probable residues (electronic supplementary material, table S1). Additionally, we performed *in silico* analysis predicting phosphosites within all the analysed lamins, supplementing the available experimental results (figure 6; electronic supplementary material, table S3).

**Table 1.** Experimentally confirmed kinases that phosphorylate lamins at verified, specified residues.

residue	lamin	organism	kinase	sequence	reference
T19	lamin A	human	Cdk1	QASS**T**PLSP	[23]
S22	lamin A	human	Cdk1	STPL**S**PTRI	[23]
S22	lamin A	mouse	Cdk5	STPL**S**PTRI	[24]
S390	lamin A	mouse	Cdk1	RLRL**S**PSPT	[25,26]
S392	lamin A	mouse	Cdk1	RLSP**S**PTSQ	[25,26]
S392	lamin A	mouse	Cdk5	RLSP**S**PTSQ	[24]
S403	lamin A	human	PKC	RGRA**S**SHSS	[27]
S404	lamin A	human	PKC	GRAS**S**HSSQ	[27]
S404	lamin A	human	Akt	GRAS**S**HSSQ	[28]
S458	lamin A (myopathy mutation)	human	Akt	LRNK**S**NEDQ	[29]
S23	lamin B1	human	Cdk1, PKC	TTPL**S**PTRL	[30]
S42/S45	lamin Dm	fly	Cdk1	QTAS**S**PLSP/SSPL**S**PTRH	[31]
S50	lamin Dm	fly	PKA	PTRH**S**RVAE	[31]
S393	lamin B1	human	Cdk5	KLSP**S**PSSR	[24]
S395	lamin B1	human	PKC	SPSP**S**SRVT	[30,32,33]
S405	lamin B1	human	PKC	SRAS**S**SRSV	[30,32,33]
S16	lamin B2	chicken	Cdk1	GTPL**S**PTRI	[34]
S16	lamin B2	chicken	MAPK	GTPL**S**PTRI	[35]
S384	lamin B2	chicken	Cdk1	RLKL**S**PSPS	[34]
S386	lamin B2	chicken	Cdk1	KLSP**S**PSSR	[34]
S400	lamin B2	chicken	PKC	ATSS**S**SSSS	[36]
S404	lamin B2	chicken	PKC	SSSS**S**TSLV	[36]
S410	lamin B2	chicken	PKC	SLVR**S**SRGK	[36]
S411	lamin B2	chicken	PKC	LVRS**S**RGKR	[36]

We also gathered data concerning particular kinases, clearly distinguishing between those confirmed experimentally (table 1) and those predicted by *in silico* analysis for detected conserved motifs. Combining experimental, large-scale and predicted data allowed us to define relationships between conservative regions, phosphosites, specific kinase motifs and function. Moreover, we demonstrated the existence of previously unrevealed clusters of phosphorylations, potential significant residues and motifs, and sites possessing crucial or potential regulatory functions, and found indications of their location in corresponding sequences of other organisms.

## 2. Sequence conservation and phosphorylation in the head domain

### 2.1. N-terminal Cdk1 site phosphorylation, related sites and kinase consensus motifs

The head domain of lamins contains at least one Cdk1 (cdc2) phosphorylation site motif, also called the lamin N-terminal mitotic site (figures 1*a* and 2). It is shared by all lamins except a single *C. elegans* lamin. The canonical phosphoacceptor serine residue is very conserved. For *H. sapiens*, it is located at position S22 in lamin A/C [23,37–39], S23 in lamin B1 [30] and S37 in lamin B2 [40]. For *X. laevis*, the positions are S18, S23, S27 and S21 for lamins A, B1, B2 and B3, respectively. For *D. melanogaster*, the positions are S37 for lamin C and S45 for lamin Dm [31,41,42]. While some nematode species have a potential N-terminal kinase motif (e.g. *Ascaris suum* has MM**SP**NRQ), Ce-lamin does not have a typical Cdk1 site [8], but the most probable mitotic site is the PKC site (S41), which is also very conserved in all lamins. *In vitro* studies demonstrated that this Cdk1 site is crucial, together with other sites, for phosphorylation-dependent polymerization and depolymerization of lamins.

This so called N-terminal (head) ‘mitotic’ site in lamins is also predicted to be an efficient target for phosphorylation by other kinases, such as Cdks (Cdk1–6), glycogen synthetase kinase (Gsk)3 and extracellular signal-regulated kinase (ERK)1–2 [43]. Experimental data indicate that this site is indeed *in vivo* phosphorylated by Cdk5, causing pathological nuclear lamina dispersion in neuronal cells [24], and by ERK1–2 kinases [35]. Moreover, when phosphorylated, this Cdk1 motif may be recognized by the Pin1 WW-binding domain [44].

The similar, conservative motif in most lamins (T19PL in human lamin A, S42PT in fly lamin Dm) also contains strong consensus sequences for Cdk5, Gsk3 and ERK1–2 kinases. Phosphorylation of this site has been discovered experimentally (figure 2; electronic supplementary material, table S1) [23,31,37,39] and in many large-scale proteomics studies (electronic supplementary material, table S2 for references).

### 2.2. Other head domain phosphorylation sites

Another interesting conserved feature in the lamin head domain is the characteristic ‘TP’ motif present in all analysed lamins except Ce-lamin, chicken lamin B1 and murine lamin B2 (figures 1*a* and 2). Its features and potential functions in the solubilization of lamin filaments can be suggested by observation of phosphorylation of this region (S5) *in vivo* on mitotic lamin A [25]. Note that the same motif (TP and less frequently SP) is also present immediately in front of the N-terminal mitotic site (figures 1*a* and 2) of all lamins, except Ce-lamin, lamin A and L3 from *Danio*, and lamin A from *Xenopus*. Phosphorylation of T3 and S5 in lamin A has been verified experimentally [39] and in large-scale proteomics studies (electronic supplementary material, table S2). Other sites that were identified as phosphorylated (but with low frequency) in large-scale proteomics studies are T3 in mouse lamin A, T3 and T5 in both human and murine lamin B1, T23 in human lamin B2, and T10 and T12 in fly lamin Dm (figure 2; electronic supplementary material, table S1). The sequences containing these sites are predicted to be phosphorylated by the kinases protein kinase (PK)A, PKC, Cdk5, Gsk3 and ERK1–2. Human lamin A has an additional predicted PKA/PKC motif, which encompasses experimentally verified phosphosites T10 and S12 [39]. It corresponds to human lamin B1 S13 (SR motif).

In fly lamin C and lamin Dm, as they have much longer head domains, there are additional phosphorylation motifs comparing with vertebrate lamins. Fly lamin Dm has the T18STPRPPS sequence, which is phosphorylated *in vivo* on S19, T20 and S25 [41,42,45–47]. S19 is predicted as a target for Gsk3, Cdk5 and ERK1–2 kinases, while T20 and S25 is presumably a target for PKC/PKA kinase. This fragment of the protein was suggested to be involved in the formation of a hairpin-like structure via interaction with the rod domain around residues of H60 and R64 [41,48]. Indeed, point mutations of these residues make lamin Dm soluble [48,49].

Another conserved head domain PKC/PKA site is located upstream of the ‘mitotic’ site. *In vitro* phosphorylation of S50 in fly lamin Dm by PKA causes depolymerization of lamin filaments [31]. In human and murine lamin B1, the corresponding phosphosites S28 and S29 were identified infrequently in large-scale proteomic studies (electronic supplementary material, table S2).

### 2.3. Conserved motifs in head domain

Detailed analyses of the head domains of various lamins within their types reveal a series of motifs specific for either A-type lamins or B-type lamins only (figure 2). The vertebrate lamin A-specific sequence METPXQK/RR differs from the lamin B-specific sequence (MATATP). In other lamins, there is no such sequence although they still have a phosphorylation site (e.g. T12 in fly lamin Dm or T23 in human lamin B2).

All lamins have predicted *in silico* phosphorylation-dependent protein-binding motifs recognized by the WW domain, mediator of DNA damage checkpoint protein 1 (MDC1) breast cancer-associated protein 1 C-terminal repeat (BRCT) domain, and polo-like kinase 1 polo-box domain (Plk1 PBD) and 14–3–3 protein [43]. METP and MATATP contain the predicted WW domain binding motif (pS/pTP; T17STP in fly lamin C and T10ATP in lamin Dm) [50,51]. This motif and other phosphorylation-dependent protein-binding motifs such as the MDC1 BRCT domain and Plk1 PBD (SpS/pTX) [43] are repeated several times in the head domain of lamins: S17ST in human and murine lamin A, S2SK, S19SP, S34TH, S41SP in fly lamin Dm.

Each lamin contains also at least one predicted 14–3–3 domain binding motif (RXXpS) [43]. This motif is located in the head domain only in lamin B1-type: R22PPS in fly lamin Dm and R10MGS in human lamin B1. Phosphorylation of both sites was experimentally confirmed (figure 2; electronic supplementary material, table S2). 14–3–3 protein is the best-known ‘interactome’ (proteome) network involved in the modulation of phosphoprotein function and location (for review see [52]). Several isoforms of 14–3–3 were reported to interact with nuclear matrix/nuclear lamina structures [28,53].

Moreover, fish and amphibian lamins B3 contain SR/RS dipeptide motifs specific for serine/arginine protein kinases (SRPKs). Such a motif is present in four copies in goldfish and torafugu, in three copies in zebrafish, and in one and two copies in *Xenopus* lamin A/B3 and B1/B2, respectively [54]. This motif is also present in invertebrates: Ce-lamin has four copies, lamin C from *D. melanogaster* has two copies and lamin Dm has a single copy. This may reflect the adaptation of lamins to the biology of organisms in which oocytes are stored in the ovaries for long periods of time in prophase (diplotene). For example, in goldfish a major component of the oocyte nuclear lamina is lamin B3, and when Cdk1 kinase is not active, a still unknown SRPK kinase phosphorylates lamin B3 at the Cdk1 site [54,55].

An interesting sequence motif is located in fly lamin C (S13RASTSTP). It is similar to the B-type lamin C-terminal motif (S/TRAS/T; figure 3) and additionally contains conserved TP residues characteristic for all lamins (except Ce-lamin). Potential functions of these motifs will be discussed later in this paper.

### 2.4. Function of the head domain

It is known that the lamin head domain is necessary for head-to-tail lamin polymerization. The direct experimental evidence of the importance of the head domain of lamins comes from *in vitro* studies. Bacterially expressed murine lamin A lacking the first 10 amino acids forms head-to-tail polymers, while removal of next 20 amino acid residues (lamin A Δ11–30) impairs polymer formation [56]. Bacterially expressed chicken lamin B2, when headless, forms dimers but fails to form head-to-tail polymers [57]. Moreover, the head domain of lamins can depolymerize the *in vitro* polymerized lamin network [58]. Also, headless fly lamin Dm fails to polymerize longitudinally [59].

As the head domain is essential for head-to-tail polymer formation, phosphorylation of sites on the head domain would keep a fraction of lamins in an unpolymerized state (dimers, tetramers) or may introduce gaps in lamin polymers facilitating lamin and lamin filament flexibility, mobility and redistribution within the nucleus. Additionally, many sites recognized by different kinases have been identified in the head domains of lamins, thus different signalling pathways may affect lamin polymerization through the head domain [60–63]. This may result in total or local nuclear lamina disassembly, or at least weakening or increased flexibility. The proteins interacting with lamins (no matter which part of the lamin they interact with) can be potentially redistributed and also made more mobile, such as that activated effector mitogen-activated protein kinases (MAPKs) may interact with lamin A at the nuclear lamina (periphery heterochromatin, transcriptionally silent genes) or inside the cell nucleus (euchromatin, potentially active genes) [35,64–66]. Thus, depolymerization and longitudinal polymerization can only be regulated by the phosphorylation state of the head domain of lamins, and they affect the lamin–proteome or lamin–chromatin network [67–69].

## 3. Sequence conservation and phosphorylation in the tail domain

### 3.1. C-terminal Cdk1 site phosphorylation, related sites and kinase consensus motifs

The tail domain of lamins contains several conserved sequences essential for all lamins [70,71] (figures 1*b*, 3–5). Proximal to the end of the rod domain lies the C-terminal ‘mitotic’ cdc2 (Cdk1) site, crucial for lamin polymerization and depolymerization (with the exception of Ce-lamin). This region contains conservative phosphoacceptor serine residues, which in *H. sapiens* are located at positions S390 and S392 in lamin A/C [23,37–39], S391 and S393 in lamin B1, and S405 and S407 in lamin B2 [40] (figure 3). In *Xenopus*, the predicted phosphoacceptor residues are S386/388, S391/393 and S395/397 for lamins A, B1 and B2, respectively. Phosphorylation of this region by Cdk1 was also confirmed experimentally for murine lamin A (S390/392) [25,26] and for chicken lamin B2 (S384/386) [34]. Near this Cdk1 site, there is a very conservative region among B1 lamins. It is also important during mitosis and may be involved in assembly and disassembly [32]. The phosphorylation site for PKC was identified in human lamin B1 (S395) [30,32,33,72]. Corresponding phosphosites for both Cdk1 and PKC were also identified in large-scale proteomics studies (figure 3; electronic supplementary material, table S1).

This canonical, conserved sequence of the C-terminal ‘mitotic’ site is not present in worm Ce-lamin and fish lamin B3. Fly lamins (Dm and C) have different sites for Cdk1 phosphorylation, and these are predicted to be modified by an entire set of kinases (Cdk5, Gsk3, ERK1–2, MAPKAPK2, PKA and PKC) [43]. In lamin Dm, the functional Cdk1 site may be located at T413 and/or the much stronger T435, which is also predicted to be phosphorylated by all Cdks (including Cdk1). This latter site was identified experimentally *in vivo* [41] and by large-scale proteomics [47]. Pseudophosphorylation of this single site significantly increases the solubility of lamin Dm [41].

### 3.2. Phosphorylation sites and binding motifs between Cdk1 site and the Ig fold

The next conserved motif in the lamin tail domain is the sequence denoted as S/TRAS/T (figures 1*b* and 3). This region contains serine residues phosphorylated by PKC, which in *H. sapiens* are located at positions S403/404 in lamin A/C [27], S405 in lamin B1 [30,32,33] and for chicken lamin B2 at S400/404 [36]. This region also contains the predicted binding motif for protein 14–3–3 [43]. Phosphorylation of the corresponding residues was identified both in site-specific experiments [40,73] and large-scale proteomics studies (electronic supplementary material, table S1). Point mutants (negative mutants and pseudophosphorylation) of this region affect the polymerization of lamins, chromatin binding and transport into the cell nucleus *in vitro* and *in vivo* [41,37]. Moreover, S404 in murine lamin A is phosphorylated by Akt, which was shown to control pre-lamin A stability and expression [28].

In *D. melanogaster*, this sequence is predicted as a target for the entire set of kinases. Note that a similar motif in fly lamin C is also located in the head domain (S13RASTST; figure 2). In vertebrates, especially mammals, the predicted candidate kinases are Akt, PKC, PKA, Gsk3 and MAPKAPK2. Phosphorylation of this motif has been demonstrated experimentally *in vivo* in single experiments [37,73] and in large-scale proteomics studies. Interestingly, this part of the tail domain contains the predicted 14–3–3 binding domain (RTPpS442) in fly lamin Dm and human lamin A (GRApS403), where the last serine is phosphorylated, and this modification was confirmed in proteomics studies (electronic supplementary material, table S1).

The detailed analyses of sequences within particular lamin types revealed additional lamin-type specific features (figure 3). A-type lamins have a less conserved and shorter consensus sequence of the C-terminal Cdk1 site than lamin B1: RLSPSP motif versus K/RLSPSPSSR, respectively. Also, the next sequences encompassing the S/TRAT/S sequence are fully conserved among B1 lamins, while lamin A proteins do not have a typical S/TRAT/S sequence and the linker region is variable. Lamins B2/B3 have the S/TRAT/S motif (except *Danio* lamin B2/B3) but within a much less conserved sequence. This suggests differences in functions of this region between lamin types.

Also, B1 lamins have an additional stretch of negatively charged amino acids immediately after the NLS, while in lamin A and lamin B2/B3 this stretch is more variable. As this region was reported to contribute to DNA/histone/chromatin binding, this may account for the differences in chromatin- and DNA-binding affinity for lamin A and lamin B (see below for discussion).

### 3.3. Ig fold

The next conserved globular region, over 105 amino acid residues in length, called the Ig fold [3], is responsible for the interaction of lamins with binding partners other than lamins (figure 4). Sequence comparison indicates that all compared lamins have this region, although the detailed structure may vary slightly between lamins. However, not all lamins have this Ig fold; for example, *Ciona intestinalis* (sea squirt) lacks it [74]. The crystal structure of the Ig fold and modification of the structure by laminopathic mutations imply that this domain may be essential for tissue-specific interaction of particular lamins with proteins [18,75,76]. Not much is known about the molecular interactions of the lamin Ig fold with binding partners. We know that laminopathic mutations localized in the Ig fold change its structure, which is in turn reflected in the properties of such lamin mutants *in vivo* [76,77].

It is also believed that the Ig fold participates in chromatin and DNA binding together with the S/TRAS/T sequence, NLS signal and polar amino acid residue stretches surrounding these sequences. Available data support this assumption, as different properties of lamin A, progerin and B-type lamins with respect to chromatin binding were reported [78–80].

Phosphorylation sites were also experimentally identified in the Ig fold, but with lower frequency. It has been reported using phosphorylation-specific antibodies that S458 in the Akt1 motif is specifically phosphorylated only in myopathy patients [29], but phosphorylation of this site has been recently demonstrated in HeLa cells [39]. Moreover, sequence R439TPS in fly lamin Dm is the predicted binding motif for the 14–3–3 domain. Besides, in the Ig fold there are potential motifs for a variety of kinases (e.g. S507 may be phosphorylated by PKA/PKC, Cdk5, Gsk3 and ERK1–2). Furthermore, S525, the phosphorylation of which is important for lamin localization in nuclear lamina during interphase [37,25], is a predicted target for PKA/PKC.

### 3.4. CaaX motif and preceding motifs

The next characteristic feature of lamins is their C-terminus with a consensus signal for farnesylation (and possible geranylgeranylation)—CaaX (figures 1*d* and 5) [4,58,81–83]. Each group of lamins has different aliphatic amino acids in this sequence (e.g. CSIM, CAIM and CYLM in human lamins A, B1 and B2, respectively). A-type lamins have the most conservative CaaX motif and the longest sequence after the Ig fold, with many identified phosphosites (figure 5). A conserved block of about 27 amino acid residues centred around LRSRTV/I may serve as a PKB/AKT phosphorylation site. Moreover, vertebrate lamin A proteins also have a signal RSYLLG for proteolytic cleavage by protease ZMPSTE24 (Face1), which probably also cleaves off the terminal aaX sequence in all A-type lamins after farnesylation [84–86]. Proximal to this site, there is a region with relatively high frequency of glycine, which suggests that this fragment of protein is not tightly ordered and is flexible. It may be involved in regulation of polymerization at the level of dimer or protofilament assembly into filaments. There is also a serine cluster (S612 up to S619 in human lamin A) which is phosphorylated *in vivo* and is predicted to be a consensus sequence for casein kinase II (figure 5; electronic supplementary material, table S1).

Next to the CaaX motif, lamins have conserved proline (-5 in lamin A and -7 in B-type lamins), which sterically helps to accommodate the farnesylation motif into the farnesyl transferase active site pocket. Although all lamins have this farnesylation motif (CaaX), experimental data on the efficiency of farnesylation of particular sequences in lamins *in vivo* are sparse. *In silico* analyses of the available experimental data analysed against the structure of the active site pocket of farnesyl transferase suggest different efficiency of farnesylation of different sequences [87]. This suggests that murine lamin B2 and B3 (CRLM motif) and human lamin B2 (CYVM) may not be efficiently farnesylated *in vivo*. If this is true, these lamins may be more easily located inside the cell nucleus than other B-type lamins.

### 3.5. Function of the C-terminal domain in lamin assembly and polymerization

Tailless lamins associate predominantly in parallel, forming dimers as building blocks. About 40 amino acid residues of the tail region (containing the C-terminal ‘mitotic’ site, ‘STRAT’ motif and NLS) are required to form a predominantly longitudinal polymer [57,59,88]. The tail domain of lamin interacts with many proteins, mostly through the Ig fold [5,20,22]. The function of the lamin tail domain in polymerization and chromatin binding will be discussed below.

## 4. Prediction of phosphorylation sites

### 4.1. Identification of phosphosites via *in silico* analysis

Limited data are available from studies of particular lamins or large-scale phosphoproteome mapping on *in vivo* identified phosphosites in lamins other than human, so we used bioinformatics tools for phosphorylation site prediction. We used three programs utilizing different algorithms: NetPhos v. 3.1 [89] DISPHOS 2 [90] and KinasePhos v. 3.10 [91]. They were chosen because they were found to be the most stringent, selective and accurate, generating the lowest number of false-negative and false-positive results when tested against fly lamins and their known phosphosites [41].

Detailed analyses of all lamin sequences detected different patterns of phosphosite location between lamins (figure 6; electronic supplementary material table S3). The bioinformatics approach demonstrated good accuracy and selectivity, comparing the results with experimentally verified *in vivo* sites in human and fly lamins. This suggests that when no experimental data are available, this approach may be useful.

Sites predicted independently with three programs (three-colour bars in figure 6) are the most likely to be phosphorylated. Our study indicated that most lamins have a high density of phosphosites on the head domain, and between the end of the rod domain and the Ig fold in the tail domain. B1 lamins and lamin Dm have phosphorylation sites in the tail domain as two clusters: one centred just after the rod domain (with the Cdk1 site) and the second centred between the NLS and the Ig fold. A-type lamins and lamin Dm proteins also have a higher density of phosphosites in the tail region after the Ig fold. Several sites are also detected on the very end of lamin A proteins in mammals. Some of the predicted sites have already been confirmed (electronic supplementary material, table S1).

When we look at the potential kinase consensus sites within the head or tail domain of lamins, we can distinguish the same kinase motifs repeated at least several times in all lamins (except Ce-lamin). The most frequently predicted phosphoacceptor sites are for PKA and PKC. The less frequently predicted sites are for Gsk-3, ERK1–2, Cdk1–5 and MAPKK. Most of the consensus sites are localized within conserved sequences, which suggests similar mechanisms of lamin regulation between different lamins and different species. Ce-lamin has many predicted PKC and PKA sites, but does not have any predicted Cdk1–5 or ERK1–2 sites and only a few sites for GSK-3 kinases, which suggests less complex regulation mechanisms of biological function of this lamin *in vivo* [43].

### 4.2. Identification of phosphosites through large-scale phosphoproteome mapping

Recently, large datasets have been made available on *in vivo* phosphorylation sites on lamins discovered during large-scale phosphoproteome mapping in various model systems. The biological samples under study are either from cell lines, normal tissues, organs, stimulated material or particular developmental stages. Although they may give us a lot of valuable data in general, they also may provide considerable false-positive data, so we graded the available records depending on how many times a particular phosphorylation site was identified, dividing the data into three groups: fewer than five datasets, 5–20 datasets and more than 20 datasets. That allowed us to indicate the most probable residues (electronic supplementary material, table S1).

Data gathered from large proteome studies proved to be valuable, and may be used to indicate particular sequences and sites for precise studies of their function. Based on this assumption, we integrated available data on lamin *in vivo* phosphorylation sites from large-scale phosphoproteome studies with individually mapped sites and illustrated them in diagrams showing common motifs in proteins. Almost all sites mapped in experiments focused on particular lamins were also confirmed via large-scale analysis. Note that the black frames in figures 2–5 mark particular phosphosites identified via large-scale proteomics studies only, whereas the pink frames mark sites mapped both individually in lamin-specific experiments and in large-scale proteomic analyses. There are some exceptions, also marked by pink frames, that were not confirmed by mass spectrometry: S5, T416, T480 and S525 in murine lamin A, or S19 and S50 in fly lamin Dm (electronic supplementary material, table S1).

### 4.3. Level of phosphorylation changes during cell cycle

We have only very limited knowledge on the effect of specified phosphorylated residues in particular lamins. Although particular phosphosites were found in many biological samples, this often does not provide conclusions on the relationship with lamin properties or function, to what extent the lamin protein was phosphorylated, how many sites were modified simultaneously on a single molecule, or which kinases were involved.

Initial studies on lamin phosphorylation attempted to assess the number of phosphate moieties per statistical lamin. For instance, the data for lamin Dm *in vivo* indicated different values ranging from 0.3 phosphates per molecule up to about three phosphates per lamin [42,45,46,92–94], and that the number of phosphates per lamin increases during mitosis [42,45,46]. A current study with phosphosite-specific antibodies indicates that the level of phosphorylation of T19, S22, S393 and S636 in lamin A/C proteins in HeLa cells during mitosis increases from twofold up to sixfold [23]. Thus, at least at the Cdk1 sites, there is strong hyperphosphorylation of lamins A/C during mitosis.

Furthermore, phosphorylation site motifs for particular kinases in lamins overlap with one another. This may lead to a situation where a particular phosphoacceptor amino acid residue in a lamin may be phosphorylated by an entire set of kinases. The best-known example of this effect is the N-terminal ‘mitotic’ consensus site and nearby sites, which can be modified not only by Cdk1, but also by other Cdks, Gsk3, ERK1–2/MAPK, PKA and PKC, and by virus kinases both *in vitro* and *in vivo* [23–26,31,32,35,54,55,95–98]. This means that multiple sites of phosphorylation can be modified by a wide range of stimuli (i.e. PKC, PKA or ERK sites can be phosphorylated by the stimulated phospholipase C pathway [98]).

Moreover, phosphorylation of a neighbouring site may have a similar effect on lamin properties *in vitro*, e.g. phosphorylation of (presumably) a ‘mitotic’ N-terminal phosphorylation site (S45) on lamin Dm by Cdk1 kinase depolymerized *in vitro* lamin Dm to the same extent as phosphorylation *in vitro* by PKC and PKA (presumably on S42 or S50) [31]. These lamin features point to the necessity of studies of function of a particular site or set of phosphosites in connection with the properties of such a lamin rather than the whole of phosphoproteome studies.

## 5. Phosphorylation and lamin properties

### 5.1. The influence of phosphorylation on polymerization

Phosphorylation of amino acid residues in the head and tail domain, especially those located next to the central coiled-coil domain, induces lamin depolymerization, might disrupt the nuclear lamina network *in vitro* and *in vivo*, and causes aberrant mitosis when mutated or pseudophosphorylated [25,34,37,38,41,96]. Typically, Cdk1 phosphorylates two ‘mitotic’ sites, one in the head and one in the tail domain. However, it seems that phosphorylation of only one of them may be sufficient for disassembly of at least some fraction of lamins [39]. It seems that A-type lamins prefer the head Cdk1 site, whereas B-type lamins prefer the tail Cdk1 site. *Drosophila melanogaster* lamin C is soluble upon single N-terminal Cdk1 site pseudophosphorylation (S37), while single C-terminal atypical Cdk1 site pseudophosphorylation in a tail fragment (T435) is sufficient for solubilization of lamin Dm [41].

Moreover, different effector kinases may trigger depolymerization of lamins during interphase and mitosis using a large variety of kinase motif sites (Cdks, PKC, PKA, Akt, ERK1–2), and lipid-mediated signalling takes part in this process [72,98]. For example, disassembly of *in vitro* assembled lamin Dm can be triggered by Cdk1, as well as by PKA [31] and human lamin B1 by PKC [96]. It is also possible that other kinases use the same Cdk1 sites for lamin disassembly or that they use different sites localized nearby [44]. For example, Cdk5 may phosphorylate Cdk1 sites in various tissues [24]. Interestingly, it is possible that nuclear lamina depolymerization depends not only on the phosphorylation of mitotic sites but also that the interphase pattern of phosphorylation must be removed [99].

Another possible effect of lamin phosphorylation may be associated with mechanical properties of the nuclear lamina as a whole or locally. It is thought that regulation of plasticity and stiffness of the cell nucleus depends on A-type lamins and their interactions with other proteins (for review see [2]). Recent experimental data indicate the direct effect of lamin A phosphorylation on particular sites affecting its polymerization on nucleoplasmic location and solubility [39,68]. It is plausible that lamin C may be possibly regulated similarly [100] as lamins A and C share most of the amino acid sequence (and phosphosites) (figure 1). Surprisingly, phosphorylation of lamin A on S22 (called mitotic site) regulates turnover of lamin proteins and assembly. Data from the same group also indicate the correlation between the stiffness of the extracellular matrix and stiffness of lamin A filaments at the nuclear envelope, and its dependence on phosphorylation [68,69,101].

These data confirm the importance of phosphorylation of lamins for location of regulatory proteins as proper phosphorylation of lamin A (and lamin C) may cause its relocation into nucleoplasm and position their interacting protein (LAP2*α*, MAPK kinases and cofactors) or vice versa [16,19,66].

### 5.2. Regulation of lamin polymerization through interactions with other proteins

Phosphorylation of either lamins in general or their specific localized fraction may affect the properties of lamins and the soluble, mobile fraction of internal lamins [39]. Moreover, longitudinal polymerization of lamins can be inhibited by lamin-interacting proteins (both phosphorylation-dependent and independent). *In vitro* lamin polymerization can be modulated by small heat shock proteins [102] or, in another example, early embryonic, soluble lamin Dm exists in a complex containing p90, p100 and p70, among others [46]. Polymerization of both bacterially expressed and purified, native lamin Dm can be inhibited by site-specific antibodies [92]. *Xenopus* lamin B3 polymerization can be inhibited by interaction with importin *α* *in vitro.* Such a mitotic fraction of lamin may participate in nucleus reassembly via RanGTP-mediated deposition of lamins at the chromatin [103].

Nuclear envelope disruption during mitotic entry is also a combination of both specific phosphorylation events leading to substantial weakening of the nuclear lamina network and tearing force generated by microtubules and motors associated with moving centrosomes and mitotic spindle machinery [104–106]. Several different protein complexes may be involved in the anchorage of spindle microtubules at the nuclear lamina. The first candidates are proteins from the LINC complex [107,108], emerin [109] and protein 4.1 [110]. Another question is the location of membranes, NE and nuclear lamina proteins near or at the mitotic spindle. Depending on the components discovered and source of the material, they are called mitotic spindle envelopes and/or spindle nuclear matrix [111–116].

### 5.3. Phosphorylation affects DNA and chromatin binding by lamins

Lamins bind directly to DNA both *in vitro* [117–121] and *in vivo* [122]. Lamins also bind to chromosomes, chromatin, nucleosomes and histones *in vitro* [123–129]. Binding to DNA and chromatin components does not require polymerization of full-length lamins (at least in the fly system); for example, lamin Dm point mutant R64H (unable to polymerize) binds chromatin and chromosomes as efficiently as wild-type lamin Dm [41] or only the tail domain of lamin Dm, which do not polymerize.

DNA and chromatin/histone-binding motifs have been demonstrated to be in the central (rod) domain [49,123] and in the tail domain [121,126–128]. Currently available data suggest that lamins interact with DNA and chromatin components mostly through conserved tail domain sequences containing conserved kinase sites for potential regulation of such interactions. A good example of such regulation is the modulation of polymerization and chromatin binding through the phosphorylation of fly lamins. Single pseudophosphorylation of fly lamin C on S37 (head Cdk1 ‘mitotic’ site) increases its solubility and mobility, and blocks polymerization, chromatin binding and reassembly of this lamin at decondensing chromosomes in the *Xenopus in vitro* nuclear assembly system [41].

It seems that the most important DNA/chromatin/histone-binding regions in B1-type lamins were mapped specifically to the sequences S/TRAT/S and NLS [41,125,127]. The S/TRAT/S sequence is present only in the B-type lamin tail domain, Ce-lamin and also in the head domain of fly lamin C (phosphorylation of S37 may affect the hairpin-like structure of lamin C and hide the chromatin-binding region S13RAST [41]). Mutation in the lamin Dm S431TRAT sequence (T432D and T435D, but also T432A and T435A) inhibited its binding to chromosomes [127]. Specific regions binding to chromatin and DNA in the A-type lamin tail domain have not been identified, but the affinity of this lamin to DNA is higher than that of lamin B1. However, the lamin A protein mutant progerin has been suggested to affect chromatin binding, indicating that the 50 amino acids that are absent in progerin may be crucial [80].

### 5.4. Phosphorylation regulates nuclear import

The mammalian lamin A sequence GRASS404 (named the Akt-binding site, which is also a target for PKC [37,25,27]) and the chicken lamin B2 PKC site S410/S411 [36] seem to be essential for nuclear import of lamins, but in contradictory manners. Pseudophosphorylation of T435 blocks nuclear import of lamin Dm [41], while mutation of S403A and S404A (lack of phosphorylation) in human lamin A also inhibits nuclear import of such lamin [37,27]. Additionally, *in vitro* phosphorylation of chicken lamin B2 by PKC inhibits nuclear import [36]. B-type lamins probably have different regulatory transport mechanisms, modulated by phosphorylation, based on the consensus sequence differences in this site in B-type and A-type lamins (S/TRAT/S versus GRASS).

Besides, phosphorylation of the chromatin-binding domain and the presence of farnesylation may also modulate the nuclear import of lamins. For example, only the double mutant of lamin Dm, T435E and the farnesylation defective, was fully cytoplasmic [41]. Nuclear import may be also modulated by phosphorylation-specific interactions with other proteins (e.g. 14–3–3 and WW domain proteins).

### 5.5. Specific and unique features of the S/TP phosphorylation motif in lamins

The S/TP motif present in lamins can be a target for both kinases and enzymes called peptidyl-prolyl isomerases (PPIs), which catalyse proline transition from *cis* to *trans* conformation, inducing structural changes in the entire polypeptide chain [130]. Only one such PPI has been discovered: specific prolyl isomerase Pin1, which is specific only for the phosphorylated S/TP motif. Typically, phosphorylation of the S/TP motif slows down spontaneous or catalysed transition of the proline residue approximately eightfold [131].

By changing the proline conformation, which may function as a ‘hinge’ site, Pin1 can enforce kinks into lamin polypeptide chains and influence lamin properties (e.g. binding to the protein partner, exposing new interaction sites, new phosphosites, etc.). Pin1 itself can also be switched off through phosphorylation (e.g. by PKA) and switched on by PP2A (protein phosphatase 2A) [132]. Recently, it was shown that Pin1 isomerase is involved in several pathways essential for cell function and signalling [133].

Direct interaction of lamin and Pin1 was reported in the case of human fibroblast infection by cytomegalovirus [44]. A small fraction of Pin1 co-localized together with a multiprotein complex containing virus kinase (pUL67), kinase-associated virus protein (pUL50) and lamin A/C at the nuclear envelope. Pin1 also co-precipitated with lamin A/C protein. Earlier studies suggested that virus kinase pUL67 and host PKC phosphorylate S22 (Cdk1 site), which then becomes the target for Pin1 and takes part in local nuclear lamina disassembly, which promotes viral nuclear egress. Indeed, lamin A S22 is phosphorylated by pUL67 kinase [134], and pS22P seems to be a Pin1-binding motif (with a WW-binding domain).

The S/TP motifs existing in lamins may function in different ways: as hinges for spontaneous or catalysed conformational changes and as part of a more complex system of regulation involving Pin1. They may keep their ‘fixed’ conformation after phosphorylation for a longer time than unphosphorylated ones. Thus, it is not phosphorylation alone, but phosphorylation in combination with active isomerases including Pin1 that may more precisely regulate lamin properties through S/TP phosphosites.

## 6. Conclusion and perspectives

The phosphorylation of lamins seems to be a major regulator of the properties of nuclear lamina. It is a crucial mechanism influencing lamin polymerization and interaction with other proteins. As there is still limited knowledge about the specific function of particular phosphosites, we attempted to indicate residues responsible for various functions and showed their importance connected with sequence conservation and the frequency of particular residue phosphorylation in different organisms and cell types. Based on the demonstrated conservation of domains with phosphosites between lamins, it is possible to draw predictions about the functions of domains and possible phosphosites in lamins that have never been tested.

The plasticity of signalling networks regulating lamin polymerization, solubility, chromatin binding, location and interactions through phosphorylation both *in vitro* and *in vivo* can be observed. Different regulatory mechanisms and different sets of kinases acting in concert are involved in the regulation of lamin polymerization, although prolonged action of single kinases or cascades (e.g. Cdk1, PKC, PKA, Cdk5, virus kinases and the lipin/phospholipase C network) may give the same effect. Potential kinases that may phosphorylate residues in consensus motifs for experimentally confirmed phosphosites were also pointed out. Their identification through *in vitro* or *in vivo* studies may reveal the plethora of signalling pathways involved in the control and modulation of lamin properties and functions.

The very intriguing aspect of lamin phosphorylation is the level of such modification in a single molecule. Lamins are hyperphosphorylated during mitosis, but the exact number of modified residues is usually not known. Very little is known about number of phosphates during interphase, despite many of them having been found. Older data suggest that phosphorylation is rather rare, that there are 0.3–3 phosphate groups per lamin molecule during interphase, and that lamins are hyperphosphorylated during mitosis. It was recently demonstrated that phosphorylation on so-called ‘mitotic sites’ increases several-fold during mitosis [40,135]. We still know very little about the real stoichiometry of phosphate on particular lamins during the cell cycle in general and about single lamins with particular properties (e.g. soluble lamin phosphate number and location versus polymerized ones *in vivo* or chromatin bound versus unbound).

Although studies on the functions of lamin phosphorylation have been conducted over the course of 35 years and a lot of data have been gathered on the location of phosphorylation sites in many model organisms, we are still far away from direct knowledge of the molecular mechanisms underlying the functions of particular sites *in vivo*. The major task of future studies in the field is to resolve the function of particular phosphosites or combinations of sites with respect to particular lamin functions and properties. We believe that a systemic *in vitro* and *in vivo* approach using pseudophosphorylation mutants and dominant negative mutants of lamins in studies directed at particular properties of lamins will prove most successful.

## 7. Data accessibility

### 7.1. Protein sequences

Protein sequences were obtained from the NCBI database (validated on 11 May 2015).

LMN_C Lamin, *Caenorhabditis elegans*; gi:17506429; ref:NP_492371.1
LC_D Lamin C, *Drosophila melanogaster*; gi:442623692; ref:NP_001260974.1
LDm_D Lamin Dm, *Drosophila melanogaster*; gi:17136290; ref:NP_476616.1
LA_F Lamin A, *Danio rerio*; gi:190337691; gb:AAI63807.1
LB1_F Lamin B1, *Danio rerio*; gi:40254675; ref:NP_694504.2
LB2-F Lamin B2, *Danio rerio*; gi:366392938; ref:NP_571077.2
LL3_F Lamin L3, *Danio rerio*; gi:42476244; ref:NP_694505.2
LA-X Lamin A, *Xenopus laevis*; gi:156119433; ref:NP_001095210.1
LB1_X Lamin B1, *Xenopus laevis*; gi:147904084; ref:NP_001080053.1
LB2_X Lamin B2, *Xenopus laevis*; gi:147901703; ref:NP_001080947.1
LL3_X Lamin L3, *Xenopus laevis*; gi:148236667; ref:NP_001081545.1
LA_G Lamin A, *Gallus gallus*; gi:45384214; ref:NP_990618.1
LB1_G Lamin B1, *Gallus gallus*; gi:45384220; ref:NP_990617.1
LB2_G Lamin B2, *Gallus gallus*; gi:45384202; ref:NP_990616.1
LA_M Lamin A, *Mus musculus*; gi:112378771; gb:ABI16251.1
LC_M Lamin C, *Mus musculus*; gi:112378773; gb:ABI16252.1
LB1_M Lamin B1, *Mus musculus*; gi:188219589; ref:NP_034851.2
LB2_M Lamin B2, *Mus musculus*; gi:113195686; ref:NP_034852.2
LB3_M Lamin B3, *Mus musculus*; gi:220472; dbj:BAA02708.1
LA_H Lamin A, *Homo sapiens*; gi:27436946; ref:NP_733821.1
LC_H Lamin C, *Homo sapiens*; gi:5031875; ref:NP_005563.1
LB1_H Lamin B1, *Homo sapiens*; gi:5031877; ref:NP_005564.1
LB2_H Lamin B2, *Homo sapiens*; gi:388240801;ref:NP_116126.3

Lamin B3 from *M. musculus* was not aligned to the other lamins because its sequence is identical to the sequence of lamin B2 from *M. musculus* in the fragments that are presented in the figures. For human lamin B2, the full sequence (with 20 ‘new’ amino acids) was used; in previous numbering M21 was M1.

### 7.2. Web resources, databases and software

Sequences were aligned with ClustalX v. 2.0, using the BLOSUM matrix and the Gonnet matrix. Each alignment was edited in Jalview v. 2.8. and individually corrected for inaccurate fragments. For each particular alignment, data from each matrix and available experimental data were taken into account. Black frames mark particular phosphosites identified in large-scale proteomics studies only, while pink frames mark sites mapped both individually in lamin-specific experiments and in large-scale proteomic studies. There are some exceptions, also marked by pink frames, that were not confirmed by mass spectrometry: S5, T416, T480, S525 in murine lamin A and S19, S50 in fly lamin Dm (electronic supplementary material, table S1). Motifs for only experimentally confirmed kinases are shown in the pictures.

Phosphorylation sites were analysed separately with three programs: NetPhos v. 2.0 server (http://www.cbs.dtu.dk/services/NetPhos), DISPHOS v. 1.3 (http://www.dabi.temple.edu/disphos/) and KinasePhos v. 2.0 (http://kinasephos.mbc.nctu.edu.tw/). NetPhos uses a method based on the neural network for predicting potential phosphorylation sites in protein sequences [89]. KinasePhos is based on the identification of protein kinase-specific phosphorylation sites, using the known phosphorylation sites, categorized by substrate sequences and their corresponding protein kinase classes [91]. DISPHOS (DISorder-enhanced PHOSphorylation predictor) uses position-specific amino acid frequencies and disorder information to identify phosphorylation sites, based on amino acid composition, sequence complexity, hydrophobicity, charge and other sequence attributes of regions adjacent to analysed phosphorylation sites (www.dabi.temple.edu/disphos). The program GenSite was used to present the phosphorylation sites in lamins (figure 6).

To predict specific motifs recognized by kinases, two programs were used: PhosphoMotif finder (http://www.hprd.org/PhosphoMotif_finder) and KinasePhos v. 2.0.

## Supplementary Material

Table S1 List of experimentally confirmed phosphosites.

## Supplementary Material

Table S2 List of representative references for phosphosites identified using mass spectrometry analysis.

## Supplementary Material

Table S3 List of predicted phosphorylated residues according to in silico analysis using three independent programs.
