# Supplementary material for: Regulation of lamin properties and functions: does phosphorylation do it all?
Source: Open Biol. 2015 Nov 18;5(11):150094. doi: 10.1098/rsob.150094 (PMC4680568; doi:10.1098/rsob.150094)
Supplement: Table S1 List of experimentally confirmed phosphosites. [file rsob150094supp1.pdf]

Supplementary Table S1. **List of experimentally confirmed phosphosites.**

Column “**Site-specific experiment**” refers to a publication on phosphorylation confirmed in a lamin-directed experiment (according to the reference list). Column “**Global MS analysis**” refers to quantity of large scale analysis (by mass spectroscopy), where lamin phosphorylation was confirmed with significant score, basing on UniProt and PhosphoSitePlus databases (+ less than 5 references; ++ 5-19 references; +++ more than 20 references). Column “**In silico analysis**” refers to a number of used programs (among: KinasePhos, NetPhos, DISPHOS) predicting particular phosphosite.

H. sapiens **Lamin A/C**

Position	Residue	Site-specific experiment	Global MS analysis	In silico analysis
3	T	[39]	+	2
5	S	[39]	+	2
10	T	[39]	+++	2
12	S	[39]	+++	0
17	S		++	1
18	S	[39]	++	1
19	T	[23,37,39]	+++	2
22	S	[23,37,38,39]	+++	2
24	T		++	0
27	T		+	2
51	S		+	3
64	S		+	1
66	T		+	0
71	S		+	2
81	Y		+	0
94	S		+	1
94	S		+	3
107	S		++	1
143	S		+	2
149	S		+	1
153	S		+	0
211	Y		+	1
212	S		++	3
224	T		+	3
277	S		++	2
295	S		+	1
301	S	[28]	+++	0
303	S		+	1
307	S		++	2
326	S		+	3
390	S	[23,39]	+++	3
392	S	[23,37,38,39]	+++	3
394	T		+++	2
395	S		+++	1
398	S		+++	2
403	S	[27,37,39]	+++	2
404	S	[27,28,37,39,73]	+++	3
406	S	[39]	+++	3
407	S	[39]	+++	2
409	T		+++	1
414	S		+++	2
416	T	[39]	++	3
423	S	[39]	++	1
424	T		++	1
426	S	[39]	++	1
429	S		++	3
431	S		+	2
437	S		+	2
458	S	[39]	+++	1
463	S		+	1
505	T		+	0
507	S		+	1
510	T		+	0
525	S	[37]	+	0
533	S		+	1
546	S		++	2
548	T		+++	3
585	T		+	0
590	T		+	0
603	S		+	2
612	S		+	1
Lamin A only				
613	S		+	0
615	S		+	2
616	S		+	1
618	S		+	1
619	S		+	3
628	S	[39]	++	1
632	S		++	2
636	S	[23,39]	++	1
643	T		+	0
652	S	[39]	++	2

Supplementary Table S1. **List of experimentally confirmed phosphosites.**

***H. sapiens* Lamin B1**

Position	Residue	Site-specific experiment	Global MS analysis	In silico analysis
3	T		+	1
5	T		++	3
13	S		+	2
19	T		++	0
20	T		+++	3
23	S	[30]	+++	2
25	T		++	0
28	S		+	3
46	Y		+	1
58	S		+	0
138	Y		+	0
158	S		+	1
200	S		++	3
210	S		++	2
212	Y		+	1
232	S		++	1
267	T		+	0
268	Y		+	0
278	S		+	2
279	S		+	2
283	T		+	0
284	S		++	1
285	T		+	0
288	S		+	2
302	S		+++	2
304	S		+	0
305	S		+	3
340	T		+	1
360	Y		+	1
375	S		++	1
391	S		+++	3
393	S		+++	2
395	S	[30,32,33]	++	2
396	S		++	0
399	T		++	3
401	S		+++	1
404	S		+++	2
405	S	[30,32,33]	+++	2
406	S		+++	1
408	S		+++	2
411	T		+	2
412	T		+	1
479	S		+	0
481	S		+	1
534	S		++	2
575	T		+++	2

***H. sapiens* Lamin B2**

Position	Former position	Residue	Site-specific experiment	Global MS analysis	In silico analysis
23	3	T		+	2
34	14	T	[40]	+++	2
37	17	S	[40]	+++	3
39	19	T		+++	0
42	22	S		+	3
79	59	S		+	2
134	114	S		+	1
168	148	S		+	1
175	155	S		+	1
246	226	S		+	2
301	281	S		+	2
316	296	S		+++	1
318	298	S		+	1
319	299	Y		+	2
374	354	Y		+	1
405	385	S	[40]	+++	3
407	387	S	[40]	++	2
409	389	S		++	2
410	390	S		+	1
418	398	T		+	3
419	399	S		+++	3
420	400	S		++	1
421	401	S	[40]	++	2
422	402	S		++	1
424	404	S		++	3
426	406	S		++	1
428	408	T		++	1
492	472	S		+	1
497	477	S		+	1
515	495	Y		+	1
541	521	S		+	2
552	532	S		+	0
556	536	T		+	0

Supplementary Table S1. **List of experimentally confirmed phosphosites.**

***M. musculus* Lamin A/C**

Position	Residue	Site-specific experiment	Global MS analysis	In silico analysis
3	T		+	2
5	S	[26]		2
10	T		+	3
12	S		++	0
17	S		++	1
18	S		+	1
19	T		++	2
22	S		+++	2
24	T		+	0
27	T		+	2
107	S		+	3
199	T	[25]		2
212	S		+	1
224	T		+	3
277	S		+	2
301	S		+++	2
303	S		+	0
326	S		+	1
390	S	[26]	+++	3
392	S	[25,26]	+++	2
394	T		++	2
395	S		++	2
398	S		+	1
403	S		++	3
404	S	[73]	++	3
407	S	[26]	+++	3
409	S	[26]	+	2
414	S		+	2
416	T	[25]		3
423	S		+	1
424	S		+	3
426	S		+	1
458	S		+++	2
463	S		+	1
480	T	[25]		3
525	S	[25]		0
533	S		+	1
546	S		++	2
548	T		++	1
Lamin A only				
570	S		++	2
573	S		++	1
575	S		+	1
587	T		+	0
614	S		+	1
617	S		+	2
619	S		+	1
620	S		+	3
629	S		++	2
633	S		++	1
637	S		++	2
644	T		+	0
646	S		+	0
647	Y		+	0
652	S	[25]	+	1
653	S		++	2
Lamin C only				
572	S	[26]		-

***M. musculus* Lamin B1**

Position	Residue	Site-specific experiment	Global MS analysis	In silico analysis
3	T		+	0
5	T		+	3
14	S		+	3
17	S		++	1
21	T		+++	1
24	S		++	3
26	T		+	1
29	S		+	3
159	S		+	2
201	S		+	3
233	S		+	1
279	S		+	2
285	S		+	1
303	S		+	2
309	S		+	0
376	S		+	1
392	S		++	2
394	S		++	3
396	S		+	1
397	S		+	1
400	T		+	3
402	S		+	1
405	S		++	1
406	S		++	3
407	S		+	0
409	S		++	3
544	S		+	1

Supplementary Table S1. **List of experimentally confirmed phosphosites.**

***M. musculus* Lamin B2**

Position	Residue	Site-specific experiment	Global MS analysis	In silico analysis
12	T		+	2
15	S		+	2
134	S		+	0
146	S		+	1
294	S		+	2
383	S		++	3
385	S		++	2
387	S		+	2
388	S		+	1
400	S		+	1
402	S		+	3
403	S		+	1
427	S		+++	3
429	S		+	1
432	S		+	2
494	T		++	1

***G. gallus* Lamin B2**

Position	Residue	Site-specific experiment	Global MS analysis	In silico analysis
16	S	[36]		2
384	S	[36]		3
386	S	[36]		2
388	S	[36]		2
389	S	[36]		0
400	S	[34]		2
404	S	[34]		3
410	S	[34]		1
411	S	[34]		2

***D. melanogaster* Lamin Dm**

Position	Residue	Site-specific experiment	Global MS analysis	In silico analysis
10	T		+	1
12	T		+	2
19	S	[46]		1
20	T		+	3
25	S	[41,42,45]	+	1
34	S		+	1
39	T		+	0
41	S		+	2
42	S	[31]	+	2
45	S	[25,41,42]	+	3
47	T		+	1
50	S	[31]		3
235	S		+	2
249	Y		+	3
250	S		+	1
311	S		+	1
413	T		+	0
435	T	[46]	+	3
442	S		+	2
455	S		+	2
459	S		+	1
595	S	[41,42,45,46]	+	3
597	T	[46]	+	1
615	S		+	0

D. melanogaster Lamin C

Position	Residue	Site-specific experiment	Global MS analysis	In silico analysis
34	S		+	3
406	S		+	3
441	S		+	3
443	T		+	3
