# Supplementary material for: Regulation of lamin properties and functions: does phosphorylation do it all?
Source: Open Biol. 2015 Nov 18;5(11):150094. doi: 10.1098/rsob.150094 (PMC4680568; doi:10.1098/rsob.150094)
Supplement: Table S2 List of representative references for phosphosites identified using mass spectrometry analysis. [file rsob150094supp2.pdf]

Supplementary Table S2. **List of representative references for phosphosites identified using mass spectrometry analysis.**

Because for some residues in lamins, there were over 200 experiments reporting phosphorylation, only the representative references were chosen (the latest and those reporting the most identified sites in lamins). For each site identified by large scale proteomic analyses, at least one reference is listed below.

***H. sapiens* Lamin A/C:**

1) Olsen JV, et al. (2010) Quantitative phosphoproteomics reveals widespread full phosphorylation site occupancy during mitosis. Sci Signal 3, ra3 Bertacchini J, et al. (2013)
2) Zhou H, et al. (2013) Toward a comprehensive characterization of a human cancer cell phosphoproteome. J Proteome Res 12, 260-71
3) Rigbolt KT, et al. (2011) System-wide temporal characterization of the proteome and phosphoproteome of human embryonic stem cell differentiation. Sci Signal 4, rs3
4) Sharma K, et al. (2014) Ultradeep human phosphoproteome reveals a distinct regulatory nature of Tyr and Ser/Thr-based signaling. Cell Rep 8, 1583-94
5) Bian Y, et al. (2014) An enzyme assisted RP-RPLC approach for in-depth analysis of human liver phosphoproteome. J Proteomics 96, 253-62
6) Kettenbach AN, et al. (2011) Quantitative phosphoproteomics identifies substrates and functional modules of aurora and polo-like kinase activities in mitotic cells. Sci Signal 4, rs5
7) Dephoure N, Zhou C, Villen J, Beausoleil SA, Bakalarski CE, Elledge SJ, et al. A quantitative atlas of mitotic phosphorylation. Proc Natl Acad Sci U S A 2008; 105:10762-7.
8) Beausoleil SA, Villen J, Gerber SA, Rush J, Gygi SP. A probability-based approach for high-throughput protein phosphorylation analysis and site localization. Nat Biotechnol 2006; 24:1285-92.
9) Moritz A, et al. (2010) Akt-RSK-S6 Kinase Signaling Networks Activated by Oncogenic Receptor Tyrosine Kinases. Sci Signal 3, ra64

***M. musculus* Lamin A/C**

1) Zanivan S, et al. (2008) Solid tumor proteome and phosphoproteome analysis by high resolution mass spectrometry. J Proteome Res 7, 5314-26
2) Wu X, et al. (2012) Investigation of receptor interacting protein (RIP3)-dependent protein phosphorylation by quantitative phosphoproteomics. Mol Cell Proteomics 11, 1640-51
3) Hsu PP, et al. (2011) The mTOR-regulated phosphoproteome reveals a mechanism of mTORC1-mediated inhibition of growth factor signaling. Science 332, 1317-22

4) Wang Z, Gucek M, Hart GW (2008) Cross-talk between GlcNAcylation and phosphorylation: site-specific phosphorylation dynamics in response to globally elevated O-GlcNAc. Proc Natl Acad Sci U S A 105, 13793-8
5) Weintz G, et al. (2010) The phosphoproteome of toll-like receptor-activated macrophages. Mol Syst Biol 6, 371 (MS)
6) Wilson-Grady JT, Haas W, Gygi SP (2013) Quantitative comparison of the fasted and re-fed mouse liver phosphoproteomes using lower pH reductive dimethylation. Methods 61, 277-86 (MS)
7) Trost M, et al. (2012) Posttranslational regulation of self-renewal capacity: insights from proteome and phosphoproteome analyses of stem cell leukemia. Blood 120, e17-27
8) Huttlin EL, et al. (2010) A tissue-specific atlas of mouse protein phosphorylation and expression. Cell 143, 1174-89
9) Villén J, Beausoleil SA, Gerber SA, Gygi SP. Large-scale phosphorylation analysis of mouse liver.P roc Natl Acad Sci U S A. 2007 Jan 30;104(5):1488-93. Epub 2007 Jan 22. (MS)
10) Sweet S.M., Bailey C.M., Cunningham D.L., Heath J.K., Cooper H.J. "Large scale localization of protein phosphorylation by use of electron capture dissociation mass spectrometry." Mol. Cell. Proteomics 8:904-912(2009)
11) Grimsrud PA, et al. (2012) A quantitative map of the liver mitochondrial phosphoproteome reveals posttranslational control of ketogenesis. Cell Metab 16, 672-83

***D. melanogaster* Lamin C**

1) Zhai B, Villen J, Beausoleil SA, Mintseris J, Gygi SP. Phosphoproteome analysis of Drosophila melanogaster embryos. J Proteome Res 2008; 7:1675-82.

***H. sapiens* Lamin B1**

1) Olsen JV, et al. (2010) Quantitative phosphoproteomics reveals widespread full phosphorylation site occupancy during mitosis. Sci Signal 3, ra3
2) Van Hoof D, et al. (2009) Phosphorylation dynamics during early differentiation of human embryonic stem cells. Cell Stem Cell 5, 214-26
3) Imami K, et al. (2008) Automated Phosphoproteome Analysis for Cultured Cancer Cells by Two-Dimensional NanoLC-MS Using a Calcined Titania/C18 Biphasic Column. Anal Sci 24, 161-6
4) Hsu PP, et al. (2011) The mTOR-regulated phosphoproteome reveals a mechanism of mTORC1-mediated inhibition of growth factor signaling. Science 332, 1317-22
5) Zhou H, et al. (2013) Toward a comprehensive characterization of a human cancer cell phosphoproteome. J Proteome Res 12, 260-71

6) Kettenbach AN, et al. (2011) Quantitative phosphoproteomics identifies substrates and functional modules of aurora and polo-like kinase activities in mitotic cells. Sci Signal 4, rs5
7) Sharma K, et al. (2014) Ultradeep human phosphoproteome reveals a distinct regulatory nature of Tyr and Ser/Thr-based signaling. Cell Rep 8, 1583-94
8) Franchin C, et al. (2014) Quantitative analysis of a phosphoproteome readily altered by the protein kinase CK2 inhibitor quinalizarin in HEK-293T cells. Biochim Biophys Acta
9) Grosstessner-Hain K, et al. (2011) Quantitative phospho-proteomics to investigate the polo-like kinase 1-dependent phospho-proteome. Mol Cell Proteomics 10, M111.008540
10) Moritz A, et al. (2010) Akt-RSK-S6 Kinase Signaling Networks Activated by Oncogenic Receptor Tyrosine Kinases. Sci Signal 3, ra64
11) Wang Z, et al. (2010) Extensive crosstalk between O-GlcNAcylation and phosphorylation regulates cytokinesis. Sci Signal 3, ra2
12) Rigbolt KT, et al. (2011) System-wide temporal characterization of the proteome and phosphoproteome of human embryonic stem cell differentiation. *Sci Signal* **4**, rs3

***M. musculus* Lamin B1**

1) Wiśniewski JR, et al. (2010) Brain phosphoproteome obtained by a FASP-based method reveals plasma membrane protein topology. J Proteome Res 9, 3280-9
2) Choudhary C, et al. (2009) Mislocalized activation of oncogenic RTKs switches downstream signaling outcomes. Mol Cell 36, 326-39
3) Huttlin EL, et al. (2010) A tissue-specific atlas of mouse protein phosphorylation and expression. Cell 143, 1174-89
4) Wilson-Grady JT, Haas W, Gygi SP (2013) Quantitative comparison of the fasted and re-fed mouse liver phosphoproteomes using lower pH reductive dimethylation. Methods 61, 277-86
5) Wu X, et al. (2012) Investigation of receptor interacting protein (RIP3)-dependent protein phosphorylation by quantitative phosphoproteomics. Mol Cell Proteomics 11, 1640-51
6) Robitaille AM, et al. (2013) Quantitative phosphoproteomics reveal mTORC1 activates de novo pyrimidine synthesis. Science 339, 1320-3
7) Trost M, et al. (2012) Posttranslational regulation of self-renewal capacity: insights from proteome and phosphoproteome analyses of stem cell leukemia. Blood 120, e17-27
8) Yu Y, et al. (2011) Phosphoproteomic analysis identifies Grb10 as an mTORC1 substrate that negatively regulates insulin signaling. Science 332, 1322-6

***D. melanogaster* Lamin Dm**

1) Zhai B, Villen J, Beausoleil SA, Mintseris J, Gygi SP. Phosphoproteome analysis of Drosophila melanogaster embryos. J Proteome Res 2008; 7:1675-82.

H. sapiens Lamin B2

1) Zhou H, et al. (2013) Toward a comprehensive characterization of a human cancer cell phosphoproteome. J Proteome Res 12, 260-71
2) Rigbolt KT, et al. (2011) System-wide temporal characterization of the proteome and phosphoproteome of human embryonic stem cell differentiation. Sci Signal 4, rs3
3) Bennetzen MV, et al. (2010) Site-specific phosphorylation dynamics of the nuclear proteome during the DNA damage response. Mol Cell Proteomics 9, 1314-23
4) Sharma K, et al. (2014) Ultradeep human phosphoproteome reveals a distinct regulatory nature of Tyr and Ser/Thr-based signaling. Cell Rep 8, 1583-94
5) Kettenbach AN, et al. (2011) Quantitative phosphoproteomics identifies substrates and functional modules of aurora and polo-like kinase activities in mitotic cells. Sci Signal 4, rs5
6) Bian Y, et al. (2014) An enzyme assisted RP-RPLC approach for in-depth analysis of human liver phosphoproteome. J Proteomics 96, 253-62
7) Franchin C, et al. (2014) Quantitative analysis of a phosphoproteome readily altered by the protein kinase CK2 inhibitor quinalizarin in HEK-293T cells. Biochim Biophys Acta
8) Grosstessner-Hain K, et al. (2011) Quantitative phospho-proteomics to investigate the polo-like kinase 1-dependent phospho-proteome. Mol Cell Proteomics 10, M111.008540
9) Olsen JV, et al. (2010) Quantitative phosphoproteomics reveals widespread full phosphorylation site occupancy during mitosis. Sci Signal 3, ra3
10) Moritz A, et al. (2010) Akt-RSK-S6 Kinase Signaling Networks Activated by Oncogenic Receptor Tyrosine Kinases. *Sci Signal* **3**, ra64
11) Bai Y, et al. (2012) Phosphoproteomics identifies driver tyrosine kinases in sarcoma cell lines and tumors. Cancer Res 72, 2501-11
12) Van Hoof D, et al. (2009) Phosphorylation dynamics during early differentiation of human embryonic stem cells. *Cell Stem Cell* **5**, 214-26

M. musculus Lamin B2

1) Tweedie-Cullen RY, Reck JM, Mansuy IM (2009) Comprehensive mapping of post-translational modifications on synaptic, nuclear, and histone proteins in the adult mouse brain. J Proteome Res 8, 4966-82
2) Choudhary C, et al. (2009) Mislocalized activation of oncogenic RTKs switches downstream signaling outcomes. Mol Cell 36, 326-39
3) Grimsrud PA, et al. (2012) A quantitative map of the liver mitochondrial phosphoproteome reveals posttranslational control of ketogenesis. Cell Metab 16, 672-83
4) Rigbolt KT, et al. (2011) System-wide temporal characterization of the proteome and phosphoproteome of human embryonic stem cell differentiation. Sci Signal 4, rs3

5) Robitaille AM, et al. (2013) Quantitative phosphoproteomics reveal mTORC1 activates de novo pyrimidine synthesis. Science 339, 1320-3
6) Wu X, et al. (2012) Investigation of receptor interacting protein (RIP3)-dependent protein phosphorylation by quantitative phosphoproteomics. Mol Cell Proteomics 11, 1640-51
7) Wilson-Grady JT, Haas W, Gygi SP (2013) Quantitative comparison of the fasted and re-fed mouse liver phosphoproteomes using lower pH reductive dimethylation. Methods 61, 277-86
8) Huttlin EL, et al. (2010) A tissue-specific atlas of mouse protein phosphorylation and expression. Cell 143, 1174-89
9) Trinidad JC, et al. (2012) Global identification and characterization of both O-GlcNAcylation and phosphorylation at the murine synapse. Mol Cell Proteomics 11, 215-29
10) Trost M, et al. (2012) Posttranslational regulation of self-renewal capacity: insights from proteome and phosphoproteome analyses of stem cell leukemia. *Blood* **120**, e17-27
