# Supplementary material for: Regulation of lamin properties and functions: does phosphorylation do it all?
Source: Open Biol. 2015 Nov 18;5(11):150094. doi: 10.1098/rsob.150094 (PMC4680568; doi:10.1098/rsob.150094)
Supplement: Table S3 List of predicted phosphorylated residues according to in silico analysis using three independent programs. [file rsob150094supp3.pdf]

Supplementary Table S3. **List of predicted phosphorylated residues according to *in silico* analysis using three independent programs.**

Phosphorylation sites were analysed separately for each lamin with three programs: NetPhos 2.0 server, DISPHOS 1.3, KinasePhos 2.0. Each residue predicted to be phosphorylated by particular program with significant probability is marked with "+" in the table and illustrated in the Fig.6. Moreover, in the Table S1, for sites confirmed experimentally, the number of programs that identified these sites by *in silico* analysis are given.

***C.elegans* Lamin C**

Position	Residue	NetPhos	KinasePhos	Disphos
2	S			+
3	S	+		
7	T		+	
9	S			+
10	S	+	+	+
14	T	+	+	+
21	S			+
22	S	+		+
24	S			+
32	S	+	+	
35	S			+
36	T	+		
41	S			+
89	S	+		
108	S	+		+
120	Y			+
153	S	+		+
185	T	+		
189	T	+		
251	Y	+		
281	Y	+		
297	S			+
312	S	+	+	
313	S	+		+
314	S		+	
318	S	+		+
325	S	+		
329	T	+	+	+
333	S	+	+	+
357	S	+		+
410	S	+		
432	Y	+		+
437	S	+	+	+
443	T			+
465	S	+		
470	S	+	+	
490	S	+		
518	Y	+		+
532	S	+		
538	S	+		+
542	T			+
544	S			+
551	S	+		+
553	S	+	+	
554	S		+	+
557	S	+	+	
564	S			+

D.melanogaster Lamin C

Position	Residue	NetPhos	KinasePhos	Disphos
2	S			+
7	T		+	
13	S	+		
16	S	+	+	+
17	T	+		
18	S			+
19	T	+	+	+
26	T	+	+	
27	S	+		+
28	S			+
33	T			+
34	S	+	+	+
37	S	+	+	
39	T			+
41	T	+		+
42	S	+		+
72	S		+	
75	T	+		
84	T			+
89	T			+
90	S	+		+
110	T	+		
139	T			+
152	Y	+		
156	Y	+	+	
163	Y	+		
214	S	+		
233	T			+
235	S	+		+
242	S	+		+
249	S	+	+	+
259	S	+		
267	Y		+	
344	T	+		
355	S		+	
406	S	+	+	+
411	T	+	+	+
412	T			+
417	S	+		+
421	S			+
426	S			+
428	S	+		
429	S	+		+
431	S	+		
435	T	+	+	
437	S	+	+	+
441	S	+	+	+
443	T	+	+	+
447	S	+		
449	S			+
457	T	+	+	
462	S	+		+
466	T			+
468	S	+	+	
470	Y		+	+
471	S			+
526	S	+		
578	S	+		
607	T	+		
616	T	+	+	+

D.rerio Lamin A

Position	Residue	NetPhos	KinasePhos	Disphos
3	T	+		+
9	S	+		+
10	S	+		
19	S	+	+	+
24	S	+	+	+
33	S	+		
48	S	+		
61	T	+		+
63	S	+		
68	S	+		+
72	S	+		+
78	Y			+
88	T	+	+	+
91	S	+		+
104	S	+	+	
110	Y	+	+	+
126	S			+
136	S			+
144	S			+
146	S			+
154	T			+
172	S	+		+
196	T	+	+	
215	S	+	+	+
219	Y	+		
221	S	+	+	
228	S	+		+
234	Y	+		+
266	S	+		
273	S			+
274	S	+		+
279	S	+		
304	S		+	
325	S	+	+	+
356	Y	+	+	+
387	S	+	+	+
389	S	+	+	+
397	T		+	+
402	S	+	+	+
404	S	+	+	
406	S	+		+
408	T			+
414	S	+		+
416	S	+	+	
418	T			+
419	S	+		+
420	S	+	+	
422	S	+		+
436	S			+
437	S	+	+	+
442	T			+
446	T			+
449	S	+	+	+
453	S	+		
455	S	+		
459	T	+	+	+
476	S	+		
495	T	+		
525	S	+	+	
528	S	+		
537	S	+		
551	S	+	+	
552	S	+	+	
562	T		+	+
564	T			+
578	S	+	+	+
579	T			+
583	S	+		
585	Y		+	+
601	S	+		
605	S	+		+
606	S	+		
609	S	+		+
611	S			+
612	S	+		+
616	S	+		+
618	S	+		
620	S	+		+
622	S	+	+	+
623	S	+		
635	S	+		

***X.laevis* Lamin A**

Position	Residue	NetPhos	KinasePhos	Disphos
3	T	+		+
10	T		+	
12	S			+
13	T	+		+
15	T		+	+
18	S	+	+	
20	T			+
23	T		+	
47	S	+		
60	T	+		
67	S	+		+
71	T	+		+
75	S	+		+
87	T	+	+	+
90	S	+		
103	S	+	+	+
121	S			+
171	S	+		+
175	T	+		
189	T	+		+
195	T	+	+	
205	S	+		
207	Y		+	
214	T	+		+
220	T	+	+	+
273	S	+		+
278	S	+		+
279	S	+	+	
291	S	+		+
297	S	+		+
303	S		+	
323	Y	+		+
329	S	+		+
330	S	+		
355	Y	+	+	+
386	S	+	+	+
388	S	+	+	
391	T	+		+
395	S			+
403	S	+		+
408	S			+
410	S			+
412	S	+	+	
422	S	+		+
424	S	+		+
425	S	+	+	
426	S	+	+	+
428	T			+
433	T			+
434	T	+		+
438	S	+	+	
448	Y	+	+	+
455	S	+		+
460	S	+		
474	T	+		+
485	T	+	+	
504	S		+	
507	S	+		
516	S	+		
529	T		+	
530	S	+		
531	S	+		
573	S	+		
574	S		+	
580	Y			+
586	T			+
591	S	+		
599	S		+	
603	S	+	+	
606	S			+
610	T			+
613	S	+		+
615	S	+		
616	S	+		+
617	S	+		
618	S			+
619	S	+	+	+
621	T			+
623	T			+
625	T	+		+
628	S	+		+
629	T			+
633	S	+		+

***G.gallus* Lamin A**

Position	Residue	NetPhos	KinasePhos	Disphos
2	S			+
3	T	+		+
5	S	+	+	
9	S	+		+
16	S			+
18	T		+	+
21	S	+	+	
26	T		+	+
50	S	+		
63	T	+		
70	S	+		+
74	S	+	+	+
90	T	+	+	
93	S	+		+
106	S	+	+	
142	S	+		
148	S			+
176	S			+
198	T	+	+	
217	T	+		+
223	T	+	+	+
238	S			+
258	Y			+
266	Y			+
276	S	+		+
281	S	+		+
282	S	+		
300	S			+
302	S	+		
306	S		+	
327	S	+	+	
358	Y	+	+	
389	S	+	+	+
391	S	+	+	
393	S			+
394	S	+		+
401	S	+		+
406	S		+	
410	S	+		+
429	T			+
430	S	+		+
437	T	+	+	
438	S	+		+
459	S	+		
481	T	+	+	+
482	Y			+
489	T	+	+	
508	S	+	+	
511	S	+		
520	S	+		
534	S	+		
574	S		+	
582	Y			+
602	S			+
608	S	+		+
610	S	+	+	
611	S		+	+
618	S			+
623	S	+		
624	S	+		+
636	S			+

***M.musculus* Lamin A**

Position	Residue	NetPhos	KinasePhos	Disphos
3	T	+		+
5	S	+		+
10	T	+	+	+
17	S			+
18	S			+
19	T		+	+
22	S	+	+	
27	T		+	+
51	S	+	+	+
64	T	+		
71	S	+		+
75	S	+	+	+
91	T	+	+	+
94	S	+		
107	S	+	+	+
121	T	+	+	+
143	S	+		+
149	S	+		
150	T			+
153	S	+		+
157	T			+
199	T	+	+	
212	S			+
218	T	+		+
224	T	+	+	+
239	S			+
259	Y			+
277	S	+	+	
282	S	+		+
295	S	+		
301	S	+		+
307	S		+	
326	S	+		
333	T	+	+	+
334	S	+		+
359	Y	+	+	+
390	S	+	+	+
392	S	+	+	
394	T		+	+
395	S	+		+
398	S	+		
403	S	+	+	+
404	S	+	+	+
406	S	+	+	
407	S	+	+	+
409	S	+	+	
414	S	+		+
416	T	+	+	+
423	S	+		
424	S	+	+	+
426	S	+		
428	S	+		+
429	S	+	+	
431	S	+	+	+
436	T			+
437	S	+		
458	S	+		+
463	S	+		
480	T	+	+	+
488	T	+	+	
507	S		+	
524	S		+	
533	S	+		
546	S	+		+
548	T	+		
570	S	+		+
573	S		+	
575	S	+		
581	Y			+
611	S	+		+
614	S			+
616	S	+	+	
617	S	+		+
619	S			+
620	S	+	+	+
622	T		+	
624	T			+
629	S	+		+
633	S	+		
637	S	+		+
652	S			+
653	S	+	+	
656	S	+	+	+
658	S	+	+	+
659	S	+		

***H.sapiens* Lamin A**

Position	Residue	NetPhos	KinasePhos	Disphos
3	T	+		+
5	S	+		+
10	T	+	+	
17	S			+
18	S			+
19	T		+	+
22	S	+	+	
27	T		+	+
51	S	+	+	+
64	T	+		
71	S	+		+
75	S	+	+	+
91	T	+	+	+
94	S	+		
107	S	+	+	+
121	T	+	+	+
143	S	+		
149	S	+		+
150	T			+
153	S	+		
157	T			+
199	T	+	+	
212	S			+
218	T	+		+
224	T	+	+	+
239	S			+
259	Y			+
277	S	+	+	+
282	S	+		
295	S	+		+
301	S	+		
307	S		+	
326	S	+		+
333	T	+	+	+
334	S	+		
359	Y	+	+	+
390	S	+	+	+
392	S	+	+	+
394	T		+	+
395	S	+		
398	S	+		+
403	S	+	+	
404	S	+	+	+
406	S	+	+	+
407	S	+	+	
409	T			+
414	S	+		+
416	T	+	+	+
423	S	+		
424	T			+
426	S			+
428	S	+		
429	S	+	+	+
431	S	+	+	
436	T			+
437	S	+		+
458	S	+		
463	S	+		
480	T	+	+	+
488	T	+	+	
507	S		+	
533	S	+		
546	S	+		+
548	T	+	+	+
572	S	+	+	
573	S	+		
579	Y			+
599	S	+		+
601	S	+		+
603	S		+	+
612	S			+
615	S		+	+
616	S	+		
618	S			+
619	S	+	+	+
621	T		+	
623	T			+
625	S			+
626	Y			+
628	S	+		
632	S	+		+
636	S	+		
652	S	+	+	
655	T			+
657	S	+	+	+

***D. melanogaster* Lamin Dm**

Position	Residue	NetPhos	KinasePhos	Disphos
2	S			+
5	S	+		+
10	T	+		
12	T		+	+
18	T			+
19	S			+
20	T	+	+	+
25	S	+		
34	S			+
35	T	+	+	+
37	S	+	+	+
41	S		+	+
42	S	+	+	
45	S	+	+	+
47	T			+
50	S	+	+	+
83	T	+		
88	T	+		
92	T	+	+	+
94	T			+
97	T	+	+	
98	T			+
118	T	+		
147	T			+
162	S		+	
171	Y	+		
202	T	+		
212	S	+		+
222	S	+		+
228	S	+		+
235	S	+	+	
241	S			+
247	T	+		+
249	Y	+	+	+
250	S	+		
257	S	+		+
258	S	+		
267	S	+	+	+
275	Y		+	
288	S	+		
304	T			+
305	S	+		+
307	S	+		
311	S	+		
317	S	+		+
382	Y			+
427	S	+		
431	S	+		+
432	T		+	
435	T	+	+	+
437	S	+	+	+
440	T	+	+	+
442	S	+	+	
455	S	+		+
459	S	+		
463	Y			+
464	Y	+		+
468	S	+		
513	T	+		
514	T		+	
515	Y	+		+
539	S	+		
545	S			+
550	S	+		
555	S	+		
566	S	+		
582	S	+	+	
586	S			+
587	S	+		
588	S			+
591	S	+		
595	S	+	+	+
597	T	+		
606	Y	+		+

D.rerio lamin B1

Position	Residue	NetPhos	KinasePhos	Disphos
7	T		+	+
13	S	+		+
15	S	+		
18	T		+	+
19	S	+		+
20	S			+
21	T		+	+
24	S	+	+	
29	S	+	+	+
50	T	+	+	
53	S	+		+
59	S		+	
77	T	+		+
81	T	+		
85	T			+
97	T	+		
113	S	+		
132	S		+	
155	S	+		+
159	T	+		+
165	S		+	
186	S	+		
201	S	+		+
220	T	+		+
226	T	+	+	+
233	S	+		+
261	Y			+
268	T	+		
269	Y	+		+
271	S	+		
279	S	+		
280	S	+		+
284	S	+		+
285	S			+
286	S	+		
288	S	+		+
297	S	+		
298	T	+		
303	S	+		+
330	S	+	+	
336	S			+
361	Y	+	+	+
392	S	+	+	+
394	S	+	+	
396	S	+		+
400	T	+	+	+
402	S			+
405	S	+		+
406	S	+	+	
407	S			+
409	S	+	+	
412	T	+		
413	T	+		+
425	S		+	+
428	S	+	+	
429	S	+	+	+
430	S	+	+	
432	S	+	+	+
436	S	+		
438	S	+		+
444	S	+		
461	S	+		
534	S	+	+	
545	T	+		+
572	Y	+		+

***X.laevis* Lamin B1**

Position	Residue	NetPhos	KinasePhos	Disphos
5	T	+		+
7	S			+
11	S	+		
12	S	+	+	+
12	T	+	+	+
16	S	+		+
17	S	+		
19	S	+		+
20	T		+	+
23	S	+		+
25	T			+
28	S	+		+
35	T		+	
37	S		+	
41	S		+	
42	S		+	
45	S		+	
50	S		+	
52	S	+		+
65	T	+		
72	S	+		+
76	S	+		
82	Y	+		
89	T	+		+
92	S	+	+	+
92	T	+	+	+
96	T	+		+
97	T		+	
108	S			+
111	S	+		+
122	S	+		+
126	S	+		
130	S			+
138	T	+		+
158	S	+		+
162	S		+	
200	S	+		+
212	Y	+		
219	T			+
221	S	+		
225	T	+		+
232	S	+		+
235	S		+	
238	Y			+
249	Y		+	
258	T	+		
260	Y			+
267	S		+	
268	Y	+		
270	S	+		
275	Y		+	
279	S	+		
283	S			+
284	S			+
287	S	+		
288	S	+		+
289	T			+
294	T	+		+
302	S	+		+
305	S	+		
308	S			+
335	S	+		+
360	Y			+
391	S	+		+
393	S	+		
395	S	+		+
396	S			+
399	T	+		+
401	S			+
404	S	+		+
405	S	+		
406	S			+
411	T	+		+
412	T	+		+
424	S	+		
427	S	+		+
428	S	+		+
429	S	+		+
431	S	+		
432	T		+	
435	S	+	+	+
435	T	+	+	+
437	S	+	+	
439	T			+
440	T		+	
442	S		+	
443	S	+		+
453	Y	+		
460	S	+		
474	T	+		
485	T	+		
488	Y	+		
514	T		+	
523	T	+		
530	T	+		
534	S	+		
543	T			+
544	T	+		+
548	T			+
565	T	+		
569	S	+		+
571	Y	+		+
582	S		+	
595	S		+	

***G.Gallus* Lamin B1**

Position	Residue	NetPhos	KinasePhos	Disphos
9	S		+	+
22	S	+	+	+
24	T			+
27	S	+	+	+
51	S	+	+	
64	S	+	+	+
75	S		+	+
83	T			+
91	T	+	+	+
95	T	+		+
125	S	+		
157	S	+	+	+
199	S	+	+	+
211	Y	+	+	
224	T			+
231	S	+		+
267	Y	+		
269	S	+		
277	S	+		
278	S	+		+
282	S	+	+	
287	T	+		
295	S	+		+
303	S	+		
304	S	+		
313	S	+		+
326	T	+		
328	S	+	+	
334	Y		+	
357	S	+		
359	Y	+	+	+
374	S			+
382	S	+		
390	S	+	+	+
394	S	+		
395	S			+
398	T	+	+	
400	S			+
403	S	+		
404	S	+	+	+
407	S	+	+	+
410	T	+		+
411	T	+		+
423	S	+	+	+
426	S	+	+	
427	S	+	+	+
428	S	+	+	
430	S	+	+	+
432	S	+		
434	S	+		+
436	S	+		
442	S	+	+	+
459	S	+		
480	S	+		
484	T	+		+
485	S	+		
487	Y	+		
507	S		+	
522	T	+		
533	S	+	+	
543	T	+		
548	T	+		+
573	S	+	+	+

***M.Musculus* Lamin B1**

Position	Residue	NetPhos	KinasePhos	Disphos
5	T	+	+	+
14	S	+	+	+
17	S	+		
21	T		+	
24	S	+	+	+
26	T			+
29	S	+	+	+
47	Y			+
53	S	+	+	
66	T	+		
77	T	+		
97	T	+		+
130	S	+	+	
159	S	+		+
177	S	+		+
201	S	+	+	+
203	T			+
213	Y		+	
220	T			+
233	S	+		
241	Y			+
279	S	+		+
280	S	+		+
285	S	+		
289	S	+		+
297	S	+		+
303	S	+		+
306	S	+	+	+
315	S			+
336	S	+		
341	S	+		+
359	S	+	+	
361	Y		+	+
376	S			+
392	S	+	+	
394	S	+	+	+
396	S	+		
397	S			+
400	T	+	+	+
402	S			+
405	S	+		
406	S	+	+	+
409	S	+	+	+
412	T	+		
413	T	+		+
425	S		+	
428	S	+	+	+
429	S	+	+	
430	S	+	+	+
432	S	+	+	
434	S	+		+
436	S	+		
438	S	+		+
461	S	+		
482	S	+		
487	S	+		
489	Y	+		
509	S		+	
524	T	+		
535	S	+	+	
544	S			+
545	T	+		+
550	T	+		+
581	S	+		+

***H.sapiens* Lamin B1**

Position	Residue	NetPhos	KinasePhos	Disphos
3	T			+
5	T	+	+	+
13	S	+		+
20	T	+	+	+
23	S	+	+	
28	S	+	+	+
46	Y			+
52	S	+	+	+
65	T	+		
76	T	+		+
96	T	+		+
126	S	+		
158	S	+		
176	S			+
200	S	+	+	+
202	T			+
210	S	+		+
212	Y	+		
225	T			+
232	S	+		
240	Y			+
278	S	+		+
279	S	+		+
284	S	+		
288	S	+		+
296	S	+		+
302	S	+		+
305	S	+	+	+
314	S			+
335	S	+		
340	T	+		
360	Y			+
375	S			+
391	S	+	+	+
393	S	+	+	
395	S	+		+
399	T	+	+	+
401	S			+
404	S	+		+
405	S	+	+	
406	S			+
408	S	+	+	
411	T	+		+
412	T	+		
424	S		+	+
427	S	+	+	
428	S	+	+	+
429	S	+	+	
431	S	+	+	+
433	S	+		
435	S	+		+
437	S	+		
460	S	+		
481	S	+		
486	S	+		
488	Y	+		
508	S		+	
523	T	+		
534	S	+	+	
543	S			+
544	T	+		+
549	T	+		+
575	T	+	+	
579	S	+		+

***D.rerio* Lamin B2**

Position	Residue	NetPhos	KinasePhos	Disphos
3	T			+
5	T	+	+	+
7	S		+	+
10	S	+	+	
14	T	+	+	+
17	S	+	+	+
22	S	+	+	+
59	S	+	+	
65	T	+		+
66	T	+		
70	S	+	+	+
74	S	+		+
90	T	+		+
106	S	+		+
145	T		+	
152	S			+
154	S	+		
194	S	+	+	+
204	S	+		
213	T	+	+	+
226	S	+		+
272	S	+		
282	T	+		
309	S	+	+	+
323	S			+
324	S	+	+	
329	Y	+		
354	Y		+	+
385	S	+	+	+
387	S	+	+	
389	S	+		+
393	T	+	+	+
395	S		+	+
397	T			+
398	T	+		
400	S	+		+
401	S	+		
402	T			+
403	S	+		+
405	S	+		
406	S	+		+
408	S	+		+
409	S	+		
421	S		+	+
431	S		+	+
439	S		+	+
441	S	+	+	
453	T		+	
458	S	+		+
463	S	+		
466	S	+		+
481	Y	+		+
484	S	+	+	
505	S	+		
507	S	+	+	
519	S	+		
533	S	+		
534	S	+		
542	T		+	+
546	S	+	+	
574	T	+		+

***D.rerio* Lamin L3**

Position	Residue	NetPhos	KinasePhos	Disphos
6	S			+
7	T	+	+	+
12	S	+	+	
14	S		+	+
20	S	+	+	+
23	S	+	+	
26	S	+	+	+
29	T			+
31	S	+	+	+
36	T	+		
66	S			+
78	S	+	+	+
79	S	+	+	+
92	S	+		
100	S	+		+
116	S		+	
169	T	+	+	
176	T	+		
229	S	+		+
233	S	+	+	+
263	T			+
286	T	+		
296	S	+		
297	T	+		
310	S	+	+	
343	S	+	+	
348	S	+	+	
368	Y	+	+	+
399	S			+
401	S	+	+	
408	S	+	+	
414	S	+		
423	T			+
434	S	+	+	+
437	S	+		
438	S			+
444	S	+	+	+
461	S	+		
475	S	+	+	
532	S	+	+	
544	Y	+	+	
545	Y	+	+	+
551	T		+	
557	S	+	+	
563	S	+		+
574	T	+	+	+

***X.laevis* Lamin B2**

Position	Residue	NetPhos	KinasePhos	Disphos
3	T			+
5	T	+	+	
7	S		+	+
9	S		+	
12	S	+		+
13	S	+		+
16	S	+	+	
22	S	+		+
23	S			+
24	T		+	+
27	S	+	+	+
29	T			+
32	S	+	+	
69	S	+	+	+
75	T	+		+
76	T	+		
80	S	+	+	+
86	Y	+		
88	S	+		
100	T	+		
122	T			+
133	S	+		+
149	S			+
162	S	+		+
175	S	+	+	
204	S	+	+	
216	Y	+	+	+
220	S	+		+
240	Y			+
242	Y	+		+
264	Y			+
282	S	+		
283	S	+		+
292	T	+	+	+
298	T			+
309	Y	+		+
333	S	+	+	
334	S	+	+	+
339	Y	+		
345	S	+		+
364	Y		+	+
395	S	+	+	
397	S		+	+
405	S			+
408	T	+	+	
409	S	+	+	+
411	S	+		+
413	S	+		
415	T		+	+
417	T	+	+	+
418	S	+	+	+
420	S	+	+	
429	Y	+		+
435	S			+
436	T		+	+
440	T		+	
442	T	+		+
446	S			+
447	S	+		
452	S	+		+
456	S	+		+
460	T			+
465	S	+		
472	S		+	
476	S		+	
482	S	+	+	+
492	Y	+		
499	S	+		
504	S	+		
522	Y	+	+	+
525	T	+		
528	Y	+		
548	S	+	+	
574	T	+		
583	T		+	+
585	T	+	+	
587	S	+		+
616	T			+
617	T	+		+
618	S	+		+
622	S	+		

***X.laevis* Lamin L3**

Position	Residue	NetPhos	KinasePhos	Disphos
3	T			+
4	S			+
5	T	+	+	
7	S	+	+	
14	S			+
18	S	+	+	+
21	S	+	+	
23	T			+
26	S	+		+
50	S	+	+	+
56	S			+
69	S	+		
70	S	+		+
74	T			+
120	S	+	+	+
142	T		+	+
152	S	+		+
173	S			+
174	S	+		+
178	T	+		
179	T			+
198	T	+	+	
223	T	+	+	
230	S	+		
238	S			+
258	Y			+
283	Y	+	+	
285	S	+		+
300	T	+		
313	S	+		
338	T	+	+	+
345	T	+		
358	Y	+		+
375	Y			+
389	S	+	+	
391	S	+	+	+
393	S	+	+	+
396	S	+		
397	T	+	+	+
399	S			+
402	S	+		
403	T			+
404	S	+	+	+
406	T	+		+
407	S			+
420	T	+		
425	T	+	+	+
428	S	+		+
429	Y			+
442	S	+		+
452	Y	+		+
464	S	+	+	
493	S	+	+	
534	S	+		
543	T	+		
545	Y	+	+	
554	T			+
572	S			+
575	S	+	+	
579	S		+	+

G.Gallus Lamin B2

Position	Residue	NetPhos	KinasePhos	Disphos
2	S			+
4	T			+
9	T	+		+
13	T		+	
16	S	+	+	
18	T			+
21	S	+	+	+
58	S	+	+	+
64	T	+		
65	T	+		+
69	S	+	+	
75	Y	+		
77	S	+		
89	T	+		
113	S	+		+
114	Y	+		
122	S	+		+
151	S	+		+
193	S	+	+	
212	T	+		+
225	T	+		
231	Y	+	+	+
295	S	+		+
297	S			+
308	S	+	+	+
310	T		+	+
320	T			+
340	T	+		+
353	Y		+	+
368	S			+
384	S	+	+	+
386	S	+	+	
388	S	+		+
392	T	+	+	+
394	S			+
397	T	+	+	+
398	S	+	+	
399	S	+		+
400	S	+		+
401	S	+		
402	S	+		+
403	S	+	+	
404	S	+	+	+
405	T			+
406	S	+	+	+
410	S	+		
411	S	+		+
424	S	+	+	
426	S		+	+
428	T			+
429	S	+		
435	S		+	+
437	S	+	+	
439	S	+		+
440	S	+		
441	S	+		+
442	S	+		
443	S	+		+
444	S	+		+
448	S	+	+	+
452	S	+	+	+
458	S	+	+	+
468	Y	+		
475	S	+		
480	S	+		
498	Y	+		
501	T	+		
504	Y	+		
522	S	+		
524	S		+	
527	S	+		
550	S	+		
559	T		+	+
561	T	+		+
563	S	+		
592	T			+
593	T	+		+
594	S	+		+

***M.Musculus* Lamin B2**

Position	Residue	NetPhos	KinasePhos	Disphos
3	S			+
12	T		+	+
15	S	+	+	
17	T			+
20	S	+	+	+
57	S	+	+	+
63	T	+		
64	T	+		+
68	S	+		
72	T	+		
74	Y	+		
76	S			+
88	T	+		
112	S	+	+	+
121	T			+
137	S			+
146	S	+		
192	S	+	+	
200	S	+		+
202	S	+		+
211	T	+	+	+
224	S	+		
225	S			+
230	Y			+
243	S	+	+	+
294	S	+		+
297	Y	+		+
307	S	+		
339	T	+		+
352	Y			+
383	S	+	+	+
385	S	+	+	
387	S	+		+
388	S	+		
391	T	+		
393	S			+
396	T	+	+	+
397	S	+	+	+
398	S	+		
399	S	+		+
400	S	+		
401	S	+	+	+
402	S	+	+	+
403	S	+	+	
408	S	+		+
421	T	+	+	
424	T	+		+
425	S	+	+	
427	S	+	+	+
429	S	+		
432	S	+		+
435	S	+		
436	S	+	+	+
438	S	+		
468	S	+		+
473	S	+		
491	Y	+		
494	T	+		
497	Y	+		
504	T	+		
517	S	+	+	
520	S	+		
521	T	+	+	
542	S	+		
556	S	+		
557	S			+
588	T			+
589	T	+		
590	S	+		+

***M.musculus* Lamin B3**

Position	Residue	NetPhos	KinasePhos	Disphos
4	S			+
6	S	+		
10	T	+		+
44	Y			+
55	T	+		
63	S			+
81	T			+
89	T	+	+	
102	S	+		+
108	Y			+
121	S	+	+	+
172	S	+		+
175	Y	+		+
185	S	+		
217	T	+		+
230	Y			+
245	S			+
261	S	+	+	
263	S	+	+	+
265	S	+		+
266	S	+		
269	T	+		
271	S			+
274	T	+	+	+
275	S	+	+	+
276	S	+		+
277	S	+		
278	S	+		+
279	S	+	+	
280	S	+	+	+
281	S	+	+	
286	S	+		+
299	T	+	+	+
302	T	+		+
303	S	+	+	+
305	S	+	+	
307	S	+		+
310	S	+		
313	S	+		+
314	S	+	+	+
316	S	+		
346	S	+		+
351	S	+		
369	Y	+		
372	T	+		
375	Y	+		
382	T	+		
395	S	+	+	
398	S	+		
399	T	+	+	
420	S	+		
434	S	+		
435	S			+
466	T			+
467	T	+		
468	S	+		

***H.sapiens* Lamin B2**

Position	Residue	NetPhos	KinasePhos	Disphos
2	S			+
5	S	+	+	
20	T			+
23	T		+	+
34	T		+	+
37	S	+	+	+
42	S	+	+	+
79	S	+	+	
85	T	+		+
86	T	+		
90	S	+	+	+
98	S			+
110	T	+		+
134	S	+		
159	S	+		+
168	S	+		
175	S			+
214	S	+	+	+
224	S	+		
233	T	+	+	+
246	S	+		+
247	S			+
252	Y			+
265	S	+	+	+
288	S	+		
292	S	+		+
293	S	+		
301	S	+		+
316	S	+		
318	S			+
319	Y	+		+
322	S			+
329	S	+	+	
361	T	+		
374	Y			+
405	S	+	+	+
407	S	+	+	
409	S	+		+
410	S			+
413	T	+	+	+
418	T	+	+	+
419	S	+	+	+
420	S	+		
421	S	+		+
422	S	+		
424	S	+	+	+
426	S	+		
428	T			+
434	S	+	+	+
450	S			+
454	T			+
469	S		+	+
471	S		+	+
473	S	+	+	+
475	S	+	+	+
492	S	+		
497	S	+		
515	Y	+		
521	Y	+		
541	S	+	+	
545	T	+	+	
553	S	+		
576	T	+	+	
580	S	+		
581	S	+		+
612	T			+
613	T	+		+
614	S	+		
618	Y	+		+
